# The multifaceted role of microRNAs in colorectal cancer: pathogenesis and therapeutic implications

**DOI:** 10.1016/j.ncrna.2025.05.012

**Published:** 2025-05-23

**Authors:** Federica Longo, Giuseppe Gattuso, Graziana Spoto, Daria Ricci, Anastasia Cristina Venera Vitale, Alessandro Lavoro, Saverio Candido, Massimo Libra, Luca Falzone

**Affiliations:** a Department of Biomedical and Biotechnological Sciences, University of Catania, Catania, I-95123, Italy; b Research Center for Prevention, Diagnosis and Treatment of Cancer, University of Catania, Catania, I- 95123, Italy

**Keywords:** microRNA, Colorectal cancer, Biomarker, Epigenetics, Therapeutic target, Drug resistance

## Abstract

MicroRNAs (miRNAs) are important regulators of gene expression and their dysregulation is involved in various diseases, including tumors. Among these, colorectal cancer (CRC) is the result of both genetic and epigenetic alterations with miRNAs playing a key pathogenetic role. Although numerous studies have investigated the most frequently dysregulated miRNAs in CRC, there is still no consensus on the specific role of individual miRNAs in the mechanisms leading to tumorigenesis, tumor progression, and the development of chemoresistance. This lack of clarity highlights the need for a deeper understanding of miRNA functions in CRC. Therefore, this review aims to clarify the role of miRNAs in CRC by examining their involvement in major oncogenic pathways, highlighting key miRNAs implicated in the disease, and exploring their potential as diagnostic biomarkers and therapeutic targets. By providing a comprehensive overview, we hope to shed light on the complex and multifaceted roles of miRNAs in CRC, which could pave the way for more effective CRC monitoring and the development of miRNA-guided therapeutic strategies.

## Abbreviations

5-Aza-2′C: 5-aza-2′-deoxycytidine
5-FU: 5-fluorouracil
ABC: ATP-binding cassette
ABCC2: ATP Binding Cassette Subfamily C Member 2
ABCF1: ATP Binding Cassette Subfamily F Member 1
ACD: Asymmetric cell division
ACOX1: Acyl-CoA Oxidase 1
ACSL1: Acyl-CoA Synthetase Long Chain Family Member 1
ACSL4: Acyl-CoA Synthetase Long Chain Family Member 4
ADAM-17: ADAM Metallopeptidase Domain 17
AFTPH: Aftiphilin
ALAS1: Aminolevulinate synthase 1
ALDH1A3: Aldehyde Dehydrogenase 1 Family Member A3
AMPKα2: Protein Kinase AMP-Activated Catalytic Subunit Alpha 2
antimiRs: anti-microRNA
AP4: Transcription Factor AP-4
APAF1: Apoptotic Peptidase Activating Factor 1
APC: Adenomatous Polyposis Coli
ASOs: Antisense oligonucleotides
ATF3: Activating Transcription Factor 3
ATG14: Autophagy Related 14
ATG5: Autophagy Related 5
ATM: Ataxia telangiectasia mutated
AXIN1: Axin 1
AXIN2: Axin 2
BAG4: BAG Cochaperone 4
BAK1: BCL2 Antagonist/Killer 1
BCL2: BCL2 Apoptosis Regulator
BCL2L1: BCL2 Like 1
BCL2L2: BCL2 Like 2
BCL9L: BCL9 Like
BIM: BCL-2-interacting mediator of cell death
BIRC5: Baculoviral IAP Repeat Containing 5
BLM: Bleomycin
BLNK: B Cell Linker
BRAF: B-Raf Proto-Oncogene, Serine/Threonine Kinase
BRG1: SWI/SNF Related BAF Chromatin Remodeling Complex Subunit ATPase 4
BTBD7: BTB Domain Containing 7
BTG2: BTG Anti-Proliferation Factor 2
BTG3: BTG Anti-Proliferation Factor 3
CA19-9: Carbohydrate Antigen 19-9
CADM2: Cell Adhesion Molecule 2
CAV1: Caveolin 1
CBR3-AS1: CBR3 Antisense RNA 1
CCND1: Cyclin D1
CCNE1: Cyclin E1
CCSCs: Colon cancer stem cells
CD133: Prominin 1
CDC42: Cell Division Cycle 42
CDCA3: Cell Division Cycle Associated 3
CDDP: Cisplatin
CDH1: Cadherin 1
CDH2: Cadherin 2
CDK19: Cyclin Dependent Kinase 19
CDK8: Cyclin Dependent Kinase 8
CDKN1C/p57: Cyclin Dependent Kinase Inhibitor 1C
CDX1: Caudal Type Homeobox 1
CEA: Carcinoembryonic Antigen
ceRNA: competing endogenous RNA
CFL1: Cofilin 1
CHD9: Chromodomain Helicase DNA Binding Protein 9
CHEK2: Checkpoint Kinase 2
CIMP: CpG island methylator phenotype
circRNA: circular RNA
CLCA4: Chloride Channel Accessory 4
CLL: Chronic lymphocytic leukemia
c-Met: MET Proto-Oncogene, Receptor Tyrosine Kinase
COX2: Cytochrome C Oxidase Subunit 2
CPA6: Carboxypeptidase A6
CRC: Colorectal Cancer
CREB1: CAMP Responsive Element Binding Protein 1
CRNDE: Colorectal Neoplasia Differentially Expressed
CSC: Cancer stem cell
CSF2R: Colony Stimulating Factor 2 Receptor Subunit Alpha
CSF2RB: Colony Stimulating Factor 2 Receptor Subunit Beta
CTSS: Cathepsin S
Cx43: Connexin-43
CXCL12: C-X-C motif chemokine ligand 12
CXCL8: C-X-C motif chemokine ligand 8
CXCR4: C-X-C Motif Chemokine Receptor 4
CXCR7: C-X-C motif chemokine receptor 7
DAB2IP: DAB2 Interacting Protein
DACH1: Dachshund family transcription factor 1
DCLK1: Doublecortin Like Kinase 1
DGCR8: DiGeorge Syndrome Critical Region 8 RNA-binding protein
DKK1: Dickkopf WNT Signaling Pathway Inhibitor 1
DKK3: Dickkopf WNT Signaling Pathway Inhibitor 3
DLC1: DLC1 Rho GTPase Activating Protein
DNAJB4: DnaJ Heat Shock Protein Family (Hsp40) Member B4
DNMT3A: DNA Methyltransferase 3 Alpha
DOT1L: Disruptor of telomeric silencing 1-like
Dox: Doxorubicin
DSB: Double-strand break
E2F3: E2F Transcription Factor 3
E2F5: E2F Transcription Factor 5
EGFR: Epidermal growth factor receptor
EIF5A2: Eukaryotic Translation Initiation Factor 5A2
ELK1: ETS Transcription Factor ELK1
EMT: Epithelial-mesenchymal transitions
EREG: Epiregulin
ERG: ETS Transcription Factor ERG
FAP: Familial Adenomatous Polyposis
FBXW7: F-Box and WD Repeat Domain Containing 7
FFPE: Formalin fixed paraffin embedded
FIP200: RB1 Inducible Coiled-Coil 1
FMNL2: Formin Like 2
FN1: Fibronectin 1
FOLFIRI: Folinic acid, fluorouracil and irinotecan
FOLFOX: Folinic acid, fluorouracil and oxaliplatin
FOXF2: Forkhead Box F2
Foxj2: Forkhead Box J2
FOXO1: Forkhead Box O1
FOXO3a: Forkhead Box O3
FRA1: FOS Like 1, AP-1 Transcription Factor Subunit
FSCN1: Fascin Actin-Bundling Protein 1
GAA: Gossypol-acetic acid
G-MDSCs: Myeloid-derived granulocyte suppressor cells
GNA13: G Protein Subunit Alpha 13
GO: Gene Ontology
GPX4: Glutathione Peroxidase 4
GRG5: Groucho-related gene 5
GSH: Glutathione
GSK3β: Glycogen Synthase Kinase 3 Beta
hCNT1: Concentrative nucleoside transporter 1
HDAC: Histone deacetylase
HDM4: Human homolog of murine double minute 4
HER2: Erb-B2 Receptor Tyrosine Kinase 2
HK II: Hexokinase 2
HMECs: Human microvascular endothelial cells
HMGA2: High Mobility Group AT-Hook 2
hnRNPA1: Heterogeneous Nuclear Ribonucleoprotein A1
HOTAIR: HOX Transcript Antisense RNA
HOXB1: Homeobox B1
HOXB3: Homeobox B3
HOXB9: Homeobox B9
HOXD10: Homeobox D10
hRFI: Human Ring-Finger homologous to Inhibitor of apoptosis protein type
HSPB2: Heat Shock Protein Family B (Small) Member 2
IGF1R: Insulin Like Growth Factor 1 Receptor
IKK-α: Inhibitor of Nuclear Factor Kappa-B Kinase Subunit Alpha
IL-17A: Interleukin 17A
IL-21: Interleukin 21
IL-6: Interleukin 6
ING4: Inhibitor Of Growth Family Member 4
IREB2: Iron Responsive Element Binding Protein 2
IRS1: Insulin Receptor Substrate 1
ITGA2: Integrin Subunit Alpha 2
JAK: Janus Kinas
JNK2: C-Jun N-Terminal Kinase 2
KDM4B: Lysine Demethylase 4B
KEGG: Kyoto Encyclopedia of Genes and Genomes
KIF14: Kinesin Family Member 14
KLF4: KLF Transcription Factor 4
KLF5: KLF Transcription Factor 5
KLK10: Kallikrein Related Peptidase 10
KRAS: Kirsten rat sarcoma
KSR1: Kinase Suppressor of Ras 1
LASP1: LIM And SH3 Protein 1
LATS2: Large Tumour Suppressor Kinase 2
LEF: Lymphoid enhancer factor
LGR5: Leucine Rich Repeat Containing G Protein-Coupled Receptor 5
LIN28A: Lin-28 homolog A
LIN28B: Lin-28 homolog B
LM: Liver metastasis
LNAs: Locked nucleic acids
LncRNA: Long non-coding RNA
LRP6: LDL Receptor Related Protein 6
LRPPRC: Leucine-rich pentatricopeptide repeat-containing protein
MACC1: MET Transcriptional Regulator MACC1
MAP4K4: Mitogen-Activated Protein Kinase Kinase Kinase Kinase 4
MAPK: Mitogen-Activated Protein Kinase 1
MAPK1: Mitogen-Activated Protein Kinase 1
MAPK7: Mitogen-Activated Protein Kinase 7
MDE: Exosomes derived from M2 macrophages
MDM2: E3 Ubiquitin-Protein Ligase Mdm2
MDR: Multidrug resistance
MDSCs: Myeloid-derived suppressor cells
MEKK: Mitogen-Activated Protein Kinase Kinase Kinase 1
MET: MET Proto-Oncogene, Receptor Tyrosine Kinase
MFN2: Mitofusin 2
MHC: Major histocompatibility complex
MIA3: MIA SH3 Domain ER Export Factor 3
MICA: MHC Class I Polypeptide-Related Sequence A
miRNA: microRNA
MK5: MAPK Activated Protein Kinase 5
MMP11: Matrix Metallopeptidase 11
MMP2: Matrix Metallopeptidase 2
MMP9: Matrix Metallopeptidase 9
MREs: microRNA response elements
mRNA: Messenger ribonucleic acid
MRP-2: Multidrug resistance-associated protein-2
MSI: Microsatellite instability
MSI-H: Microsatellite instability high
MSS: Microsatellite status
MST3: Mammalian STE20-Like Protein Kinase 3
mTOR: mammalian target of rapamycin mTOR
MUC1: Mucin 1
MVs: Microvesicles
MYO6: Myosin VI
NAMPT: Nicotinamide Phosphoribosyltransferase
ncRNA: non-coding RNA
NEAT1: Nuclear Paraspeckle Assembly Transcript 1
NEDD9: Neural Precursor Cell Expressed, Developmentally Down-Regulated 9
NF2: Neurofibromin 2
NF-κB1: Nuclear Factor Kappa B Subunit 1
NM23-H1: NME/NM23 Nucleoside Diphosphate Kinase 1
NOTCH3: Notch Receptor 3
NPEPL1: Aminopeptidase Like 1
NRP1: Neuropilin 1
NT5E: 5'-Nucleotidase Ecto
OAZ2: Ornithine decarboxylase 2
OCLN: Occludin
OCT4: Octamer-binding transcription factor 4
P130: RB Transcriptional Corepressor Like 2
PAK4: P21 (RAC1) Activated Kinase 4
PBX3: PBX Homeobox 3
PDCD4: Programmed Cell Death 4
PDE4D: Phosphodiesterase 4D
PDH: Pyruvate Dehydrogenase
PDK1: Pyruvate Dehydrogenase Kinase 1
PDK4: Pyruvate Dehydrogenase Kinase 4
PD-L1: Programmed Death Ligand 1
PFN2: Profilin 2
PGE2: Prostaglandin E2
PI3K: Phosphatidylinositol 3-kinase
PIAS3: Protein Inhibitor Of Activated STAT 3
PLCD1: Phospholipase C Delta 1
PPAR: Peroxisome Proliferator Activated Receptor Alpha
PPP2R5E: Protein Phosphatase 2 Regulatory Subunit B'Epsilon
PRRX1: Paired Related Homeobox 1
PTBP1: Polypyrimidine Tract Binding Protein 1
PTEN: Phosphatase And Tensin Homolog
PTK6: Protein Tyrosine Kinase 6
PTP4A: Protein Tyrosine Phosphatase 4A
PUMA: p53 upregulated modulator of apoptosis
RAC1: Rac Family Small GTPase 1
RANBP1: RAN binding protein 1
Ran-GTPase: nuclear RAS-related protein-guanosine-5'-triphosphate-ase
RAP1B: RAP1B, Member Of RAS Oncogene Family
RASA1: RAS P21 Protein Activator 1
RBL2: RB transcriptional co-repressor like 2
RCN2: Reticulocalbin 2
RECK: Reversion Inducing Cysteine Rich Protein With Kazal Motifs
RFFL: Ring Finger and FYVE Like Domain Containing E3 Ubiquitin Protein Ligase
RISC: RNA-induced silencing complex
RMST: Rhabdomyosarcoma 2 Associated Transcript
RND3: Rho Family GTPase 3
RNF6: Ring Finger Protein 6
ROS: Reactive Oxygen Species
RPL11: Ribosomal Protein L11
RPS15A: Ribosomal Protein S15a
RUNX3: RUNX Family Transcription Factor 3
SATB2: SATB Homeobox 2
SCD: Stearoyl-CoA Desaturase
SCD: Symmetrical cell division
SEMA6D: Semaphorin 6D
SFRP4: Secreted frizzled-related protein 4
shRNAs: short hairpin RNAs
SIP1: SMAD Interacting Protein 1
siRNA: small interfering RNA
SIRT1: Sirtuin 1
SIRT4: Sirtuin 4
SM: Small molecule
SMAD3: SMAD Family Member 3
SMAD4: SMAD Family Member 4
SMAD7: SMAD Family Member 7
SMIR: Small inhibitors of miRNA
SNAIL: Snail Family Transcriptional Repressor 1
SOCS1: Suppressor of cytokine signaling 1
SOCS3: Suppressor Of Cytokine Signaling 3
SOX2: SRY-Box Transcription Factor 2
SOX4: SRY-Box Transcription Factor 4
SOX5: SRY-Box Transcription Factor 5
SPINT1: Serine Peptidase Inhibitor, Kunitz Type 1
SPOP: Speckle Type BTB/POZ Protein
SRC: SRC Proto-Oncogene, Non-Receptor Tyrosine Kinase
SRCIN1: SRC kinase signaling inhibitor 1
SSH2: Slingshot Protein Phosphatase 2
ST6GALNAC2: ST6 N-Acetylgalactosaminide Alpha-2,6-Sialyltransferase 2
STAT: Signal Transducer and Activator Of Transcription
TAMs: Tumor-associated macrophages
TBPL1: TATA-Box Binding Protein Like 1
TCF: β-catenin-T cell factor
TCF4: Transcription Factor 4
TCGA: The Cancer Genome Atlas
TEAD4: TEA Domain Transcription Factor 4
TGFB2: Transforming Growth Factor Beta 2
TGFBR2: Transforming growth factor receptor β
TGFβ: Transforming Growth Factor Beta
THBS1: Thrombospondin 1
THBS2: Thrombospondin 2
TIAM1: TIAM Rac1 Associated GEF 1
TICs: Tumor-initiating cancer stem cells
TNFAIP3: TNF Alpha Induced Protein 3
TNF-α: Tumor Necrotic Factor Alpha
TP53INP1: Tumor Protein P53 Inducible Nuclear Protein 1
TPM1: Tropomyosin 1
TSA: Trichostatin A
TSP-1: Thrombospondin 1
TYMS: Thymidylate Synthetase
VAPA: VAMP Associated Protein A
VCR: Vincristine
VEGFA: Vascular Endothelial Growth Factor A
VIM: Vimentin
VLDLR: Very Low Density Lipoprotein Receptor
VOPP1: Vesicular pro-survival protein 1
WDR43: WD Repeat Domain 43
WIF1: WNT Inhibitory Factor 1
XIAP: X-Linked Inhibitor of Apoptosis
XIST: X Inactive Specific Transcript
XPO5: exportin5
YAP1: Yes-associated protein 1
YES: YES1 Proto-Oncogene, Src Family Tyrosine Kinas
ZBTB2: Zinc finger and BTB domain containing 2
ZEB1: Zinc Finger E-Box Binding Homeobox 1
ZEB2: Zinc Finger E-Box Binding Homeobox 2
ZNF281: Zinc Finger Protein 281
ZNRF3: Zinc And Ring Finger 3

## 1 Introduction

Colorectal cancer (CRC) is the fourth most common cancer and the second leading cause of cancer-related deaths globally. The incidence of CRC varies significantly between geographical areas [1]. Age is one of the main risk factors for CRC development; however, CRC incidence rates have decreased by up to 50 % in older age groups in the US as a result of screening programs [2]. According to Vogelstein's model, truncating mutations affecting the adenomatous polyposis (*APC*) gene play a crucial role in the regulation of cell adhesion and proliferation due to the alteration of the Wnt/β-catenin axis actively involved in the formation of adenomatous polyps. Following *APC* mutation, the progression of CRC involves a series of additional genetic changes, notably mutations in *KRAS* and *TP53* involved in cell growth and differentiation and the loss of cell cycle control and increased mutation rates, respectively [3,4]. Serrated polyp pathway is an alternative pathway that leads to CRC development, characterized by the presence of serrated lesions that can give rise to colorectal malignancies. The most frequent initiating event in this pathway is *BRAF* mutation which promotes cell proliferation and survival. This first molecular trigger leads to extensive methylation of CpG islands, leading to the silencing of critical tumor suppressor genes and further tumor-promoting events. Hypermethylation often affects the promoter region of genes coding for mismatch repair proteins, resulting in a deficiency of the mismatch repair enzyme. These tumors are defined as CIMP+ (CpG island methylator phenotype). Besides CIMP, other typical CRC phenotypes are chromosomal instability (CIN), involving several numerical chromosome aberrations, and microsatellite instability (MSI) CRC [5]. Understanding the molecular mechanisms underlying the development of CRC is essential as it provides the basis for current screening strategies or to predict the prognosis of patients. In this context, several tests, both non-invasive and invasive, are used for CRC screening, however, the diagnosis of CRC is only obtained by histopathological examination [6]. In daily clinical practice, several biomarkers have been proposed for the early detection of CRC and to monitor the disease. Among these, the most relevant include CEA and CA19-9, which have a good predictive value for the monitoring of the disease but have a low specificity and sensitivity for CRC diagnosis [7]. Other studied biomarkers are different antibodies, circulating mutations, specific aberrant RNA transcripts and epigenetic biomarkers, including microRNAs (miRNAs). Concerning CRC therapy, the most curative intervention still relies on surgery for the treatment of localized CRC. Chemotherapy, using 5-fluorouracil (5-FU), capecitabine, irinotecan, oxaliplatin and folic acid (FOLFOX/FOLFIRI regimens) is mainly used in the adjuvant setting after surgery, or as a neoadjuvant treatment to shrink the tumor mass before surgical treatment, especially in rectal cancer and some colon tumors. In addition, these drugs can also be administered in combination with radiotherapy or with immunotherapy in microsatellite instability (MSI) CRC [8,9]. In addition to standard chemotherapy, the detection of specific mutations in the *RAS* (KRAS) and *BRAF* genes is important to consider patients eligible for targeted therapy [10]. In the case of wild-type *KRAS* and *BRAF* genes, patients can benefit from the FOLFOX/FOLFIRI protocol combined with the anti-EGFR selective inhibitor, named cetuximab [11]. Finally, immunotherapy based on immune checkpoint inhibitors has proven effective for the treatment of metastatic CRC with high microsatellite instability (MSI-H) [12]. Despite the multiple therapeutic options currently available, drug resistance mechanisms may lead to therapeutic failure, affecting the prognosis of patients [13]. In this heterogeneous molecular context, mounting scientific evidence is demonstrating the tumorigenic role of non-coding RNA (ncRNA) in CRC, with miRNAs playing a key role in tumor invasion, metastasis, and chemoresistance. Despite numerous studies, the specific roles of individual miRNAs remain to be fully elucidated, making them a focal point for future CRC research aimed at improving personalized medicine strategies and predicting the development of drug resistance [14,15].

## 2 microRNAs biogenesis, function and role in cancer

miRNAs are a class of ncRNAs short in size (19–25 nucleotides) that play important roles in regulating the expression of homologous target-gene transcripts through a mechanism known as RNA interference (RNAi) [16]. The biogenesis of miRNAs is a multi-step process that begins in the nucleus, where a long primary transcript (pri-miRNA) is produced and then processed into a precursor miRNA (pre-miRNA) by a multi-protein complex consisting of the DiGeorge syndrome critical region 8 RNA-binding protein (DGCR8) and the ribonuclease III enzyme, Drosha [17]. Subsequently, at the cytoplasmic level, the pre-miRNA is cleaved by the RNase III Dicer endonuclease to form a miRNA duplex, of which one strand will be loaded into the RNA-induced silencing complex (RISC) [18]. Usually, miRNAs interact with the 3′ untranslated region (3′ UTR) of the targeted mRNA to induce mRNA degradation or translational repression. However, miRNAs interacting with other mRNA regions (5′ UTR, coding sequence, and gene promoters) have also been reported [19]. Since a single miRNA can target hundreds of mRNAs and a single target mRNA can be silenced by several miRNAs, the understanding of this epigenetic regulatory network is very intricate and requires high-throughput platforms. miRNAs regulate several biological processes, including oncogenic or tumor suppressor pathways. Therefore, miRNAs aberrant expression can contribute to the development of several pathological conditions including cancer [[20], [21], [22], [23], [24], [25], [26]]. The link between miRNA alteration and cancer development was first demonstrated by Croce and colleagues in 2002. Specifically, Croce's study showed a deletion of the miR-15a/16-1 cluster in chronic lymphocytic leukemia (CLL) associated with tumor progression, thus suggesting the tumor suppressor role of these miRNAs [27]. After this pivotal study, several researchers investigated the role of miRNAs in cancer pathogenesis.

### 2.1 microRNAs as targets for therapeutic application

**Fig. 1:**
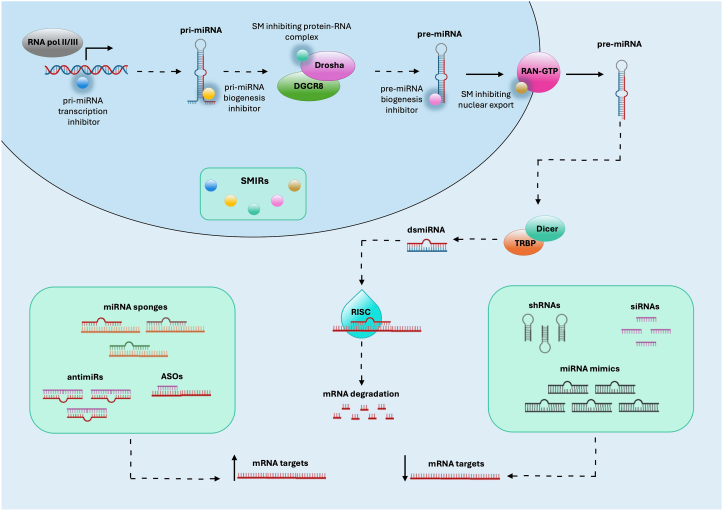
Several strategies are available to modulate the levels of miRNAs in the cell. Small miRNA inhibitors (SMIRs) can inhibit the activity of miRNAs by hindering steps in their biogenesis. Antisense oligonucleotides (ASOs), anti-microRNA ASOs (antimiRs) or miRNA sponges are molecules that inhibit miRNA function while small interfering RNAs (siRNAs), short hairpin RNAs (shRNAs) or mimic miRNAs are molecules that mimic miRNA activity.

Recently, miRNAs have emerged as attractive targets for therapeutic application. Different miRNA-targeting strategies have been implemented mainly using small molecules (SM) and small inhibitors of miRNAs (SMIRs) (Fig. 1). Among the SM, molecules inhibiting pri-miRNAs, pre-miRNAs, or protein-RNA complexes were developed [28]. An example of SM in Enoxacin that belongs to the family of synthetic antibacterial compounds with a fluoroquinolone skeleton, which enhances RNAi induced by shRNA or siRNA duplexes [29]. Among SM, a subclass is defined as SMIRs since they are able to inhibit miRNA activity. In contrast to oligonucleotide-based therapies targeting mRNAs or miRNAs, SMIRs represent an innovative and promising therapeutic strategy due to their better cellular uptake capacity, greater stability, and the possibility of being administered orally [28]. Examples of SMIRs are azobenzene-2 [30], Targaprimir-96 [31], Benzimidazole [32], Targapremir-210 [33] or AC1MMYR2 [34]. Moreover, bifunctional chimeric molecules obtained by the fusion of a recognition module with a proteolysis-targeted RNA degradation module (ProTaC) have been developed to hinder miRNA biogenesis. For instance, bleomycin (BLM), a natural compound known for its RNA-cleaving properties, was fused with specific ligands to target pri-miR-96, thus allowing specific RNA cleavage and degradation [35]. In addition, several RNA-based therapies have been developed, including antisense oligonucleotides (ASOs), anti-microRNA (antimiRs), small interfering RNAs (siRNAs), short hairpin RNAs (shRNAs), miRNA mimics, miRNA sponges, therapeutic circular RNAs (circRNAs) and CRISPR/Cas9-based gene editing [36] (Fig. 1).

miRNA mimic molecules are synthetic double-stranded RNA oligonucleotides used for cell transfection. At the cellular level, miRNA mimics are transformed into the single-stranded form by regulating the expression of target genes through a miRNA-like function [37].

Inhibitors of miRNAs, or anti-miRs, bind selected miRNAs by blocking their function [38]. A major problem associated with the use of miRNA inhibitors is their limited half-life. Indeed, naked nucleic acids are susceptible to degradation by nucleases. Another issue is related to targeted delivery, as these nucleic acids must be transported into the cytoplasm or into the nucleus to perform their function [39,40]. Based on these challenges, several chemical modifications of nucleic acids have been developed to resist nuclease degradation, reduce immunogenicity and improve miRNA-target interactions [38,41].

Among miRNA inhibitors, antagomirs are synthetic cholesterol-conjugated RNAs with a 2′-O-methyl bond and a phosphorothioate modification. However, antagomirs require high dosages to effectively block miRNAs [42]. Furthermore, most miRNA-based therapeutic agents employ other modifications, such as locked nucleic acids (LNAs) [43,44].

miRNA sponges are RNAs designed to carry multiple tandem binding sites complementary to a heptamer in the seed sequence of the miRNA of interest. Consequently, a single sponge type can block an entire miRNA seed family. However, for the same reason, miRNA sponges could lead to off-target effects; they also have a relatively low binding affinity and the concentration required to effectively block miRNA function is higher than LNA or antagomir [45].

Ultimately, other possible therapeutic applications of miRNA targeting can be focused on the inhibition of oncogenic miRNAs by using miRNA antagonists [46] or restoring miRNA expression using a tumor suppressor mimic miRNA to recover its loss of function [47]. The efficacy of miRNA- and siRNA-based therapies has been tested in several clinical trials. Examples are TargomiR (miR-16 mimic-based therapy) used for the treatment of mesothelioma, Cobomarsen (anti-miR-155) in T-cell leukemia/lymphoma, and Miravirsen (anti-miR-122) in individuals with hepatitis C infection. Nusinersen (Spinraza), is a fully MOE-modified 18-mer ASO that redirects the splicing of *SMN2* gene, approved for the treatment of spinal muscular atrophy [48,49]. An example of siRNA-based drug is Onpattro (Patisiran) containing 2′-*O*-methyl modified and unmodified ribonucleosides, with 2′-deoxythymidine dinucleotide overhangs at the 3′ ends, which is encapsulated in lipid-based nanoparticle approved by the FDA for the treatment of amyloidosis, marking a significant milestone in the history of RNAi technology and establishing a new therapeutic class [40,50]. Givosiran (Givlaari™) is another siRNA-based drug that targets aminolevulinate synthase 1 (*ALAS1*) and is covalently linked to a ligand that drives it into hepatocytes. Downregulation of *ALAS1* mRNA by Givosiran prevents the accumulation of neurotoxic δ-aminolevulinic acid and porphobilinogen both responsible for acute porphyria attacks [51].

The identification and targeting of the mainly dysregulated miRNAs in CRC that play a crucial role in cancer progression and the development of chemoresistance is of major importance. Therefore, miRNA- and siRNA-based therapies may represent an innovative therapeutic approach with the potential to improve treatment outcomes and to overcome drug resistance.

## 3 Role of microRNAs in colorectal cancer

As previously mentioned, the alteration of miRNA expression levels may depend on several factors. Tumorigenesis is often accompanied by chromosomal aberrations such as deletions, amplifications or translocations. In many cases, miRNA alterations result from a variation in the copy number of specific genomic loci. Other factors that can impact miRNA expression are epigenetic modifications. DNA methylation plays a key role in regulating the expression of suppressor miRNAs in cancer cells. The hypermethylation of the promoters of let-7, miR-34, miR-342, miR-345, miR-9, miR-129, and miR-137 was associated with a reduced expression of these miRNAs and consequently with CRC development [52]. The hypermethylation of CpG islands in miRNA coding promoter regions results in their transcriptional silencing as demonstrated in CRC models for the miR-143, miR-145, and miR-133b. In particular, histone acetylation and DNA methylation were investigated in early and late-stage CRC cells (SW1116 and DLD1, respectively) by treatment with 5-aza-2′-deoxycytidine (5-Aza-2′C) and the histone deacetylase (HDAC) inhibitor trichostatin A (TSA). The epigenetic modulation of miRNA expression induced by these treatments demonstrated that miRNA expression is sensitive to DNA demethylation in both early- and late-stage CRC cells, whereas histone acetylation has a moderate influence on miRNA expression only in early-stage CRC [53].

The altered expression of miRNAs may be related to the impaired activity of transcription factors that regulate the transcription of pri-miRNAs [54]. Another mechanism that may affect the expression of miRNAs is mediated by competing endogenous RNA (ceRNAs). Salmena and colleagues in 2011 first formulated the ceRNA hypothesis, according to which there is a cross-talk between both coding and non-coding RNAs via microRNA response elements (MREs) whose alteration could influence disease onset [55]. This category of ncRNAs includes circRNAs and lncRNAs. CircRNAs are a class of closed-loop RNA molecules that play an important regulatory role in modulating miRNA functions through sponge adsorption. Besides sponging, the circRNA-miRNA interaction mechanisms also include storage and transport of miRNAs and the interference with their expression. LncRNAs can also sponge miRNAs and compete with them for interaction with mRNA [56,57]. For instance, HOX transcript antisense intergenic RNA (HOTAIR) negatively regulates the expression of miR-203a-3p, miR-545 and miR-218, leading to the up-regulation of their targets, such as β-catenin, groucho-related gene 5 (*GRG5*), epidermal growth factor receptor (*EGFR*), and vesicular pro-survival protein 1 (*VOPP1*), involved in the proliferation of CRC cells [[58], [59], [60]]. In addition to the endogenous “sponge” effect, lncRNA-miRNA interaction can accelerate miRNA degradation [61]. In the context of CRC, miRNAs can also play a role in physiological and pathological processes by influencing cancer stem cell biology and angiogenesis, epithelial-mesenchymal transitions (EMT), and drug resistance [62,63]. The expression of miRNAs can be altered at different tumor stages, including tumor initiation, progression and metastasis. Specific tumor histotypes have a distinct signature of altered miRNAs compared to matched normal tissue and other tumors. When altered, miRNAs promote tumor progression by affecting the mechanisms of cell growth, cell motility, alteration in hormonal stress response, proliferation, evasion of tumor suppression, apoptosis, metastasis, angiogenesis and drug resistance [64].

A comprehensive assessment of differentially expressed miRNAs and target genes between CRC samples and healthy controls may facilitate the identification of miRNAs functionally related to this tumor. Specific CRC miRNA expression profiles have been identified through several differential analysis studies. Such studies are generally based on miRNA profiling performed on different sources of samples (cells, fresh tissue, formalin-fixed paraffin-embedded tissue, body fluids), analyzed by using RT-qPCR panels, microarray or RNA sequencing platforms. After wet analyses, the data obtained are processed by using both statistical and bioinformatics approaches [65]. A RT-qPCR study analyzing isolated colonic crypts from 24 CRC patients identified 13 differentially expressed miRNAs in tumor glandular cells and surrounding stromal cells. Specifically, miR-130a-3p, miR-143-3p, miR-206, miR-31-5p, miR-27a-3p and miR-27b-3p were found to be upregulated in gland cells isolated from CRC compared to non-tumor samples, while miR-21-5p, miR-195-5p, miR-19a-3p, miR-34b-3p, miR-186-5p, miR-191-5p and let-7a-5p were downregulated [66]. Another microarray and RT-qPCR study comparing miRNA expression levels in 12 CRC tissue samples and 9 adjacent normal tissues found that miR-31 was significantly upregulated in CRC. Notably, this miRNA plays a significant role in activating the RAS signaling pathway by inhibiting the translation of *RASA1*, thereby increasing the growth of CRC cells and promoting tumorigenesis [67]. Likewise, miR-31, together with miR-18a and miR-21-5p, was identified among the most upregulated miRNAs associated with *APC* gene alterations by another differential expression study conducted on 40 CRC tumor samples and in their paired normal counterpart. In this study, it was found that miR-31 expression levels correlate with the expression of the tumor biomarker CA19-9 [68]. Using miRNA-Seq, Shaath H et al., performed a miRNA expression profiling on 15 CRC tissues compared to the corresponding normal adjacent mucosa. *miR-133a-3p*, *miR-363-3p*, *miR-145-5p*, and *miR-195-3p*, were found to be the most downregulated miRNAs while *miR-135b-5p*, *miR-552-5p*, *miR-224-5p*, *miR-183-5p* and *miR-552-3p* were found among those miRNAs upregulated in CRC [69]. Almeida MI et al., evaluated the expression levels of miR-28-5p and miR-28-3p in 108 CRC and 49 normal colorectal samples, of which 47 were paired, finding that both miRNAs were downregulated in CRC compared to normal tissues. They also conducted analyses on HCT116, RKO and SW480 cells, demonstrating how miR-28-5p restoration altered the expression of *CCND*1 and *HOXB3* and reduced the proliferation, migration and invasion of CRC cells, while miR-28-3p modulated the expression of *NM23-H1* and increased the migration and invasion of CRC cells *in vitro* [70]. Ling H et al. performed a study based on miRNA microarray profiling in primary CRC tissues of patients with (N = 4) and without (N = 8) metastases. In particular, the expression of miR-224 increased with a positive correlation with tumor burden and microsatellite stability status. *SMAD4*, a target of miR-224, shows a negative correlation with miR-224 expression in clinical samples. Thus, miR-224 might, in part, promote CRC metastasis through the regulation of *SMAD4* [71]. Another differential expression analysis based on microarray showed that miR-139 is downregulated in 34 CRC tissues compared with corresponding noncancer tissues. Restoration of miR-139 did not inhibit CRC cell growth but suppressed CRC cell metastasis and invasion *in vitro* and *in vivo* by inhibiting the IGF-IR/MEK/ERK axis and down-regulating the matrix metalloproteinase 2 (*MMP-2*) [72]**.**

In addition to these experimental studies, several bioinformatics investigations based on the integrated analysis of multiple profiling data were performed to establish CRC-associated signatures. For instance, Jevšinek Skok D et al. analyzed the high-throughput molecular profiling data of 295 CRC samples from The Cancer Genome Atlas (TCGA) database. The genes *FN1*, *TGFB2*, *RND3*, *ZEB1* and *ZEB2* and the miRNAs miR-200a/b/c-3p, miR-141-3p and miR-429 were selected as the most associated with CRC, while a negative correlation was found between the miRNA miR-200b/c-3p and its target gene *FN1* and between miR-200a-3p and its target *TGFB2* [73]. Falzone L et al., analyzed the miRNA expression levels observed in CRC samples and normal tissues from different miRNA microarray expression datasets obtained from the Gene Expression Omnibus DataSets database. In this analysis, 19 differentially expressed miRNAs were identified. In addition, it was shown that the up-regulated miRNAs miR-183-5p and miR-21-5p and the down-regulated miRNAs miR-195-5p and miR-497-5p play important roles in the regulation of the mismatch repair mechanism as well as in the Wnt, RAS, MAPK, PI3K, TGF-β and p53 signaling pathways involved in the development and progression of CRC [74]. A bioinformatics study further identified 874 targets for tissue-specific miRNAs and 157 for circulating miRNAs most frequently altered in CRC. In particular, this analysis showed that miR-424-5p, miR-96-5p, miR-1290, miR-224, miR-133a and miR-363-3p target genes known to play a role in CRC, including *BRAF*, *KRAS*, *EGFR*, *APC*. Moreover, miR-133a and miR-96-5p regulate the PI3K-AKT signaling pathway, which is known to be associated with CRC progression [75]. The data obtained on miR-133a were also confirmed in other tumors of the gastrointestinal tract, including oral cancer and gastric cancer [76,77].

In all mammals, several miRNAs are organized in genomic clusters on single polycistronic transcripts containing two or more miRNAs with similar sequences. Usually, a cluster corresponds to a single transcriptional unit; thus, the members of a miRNA cluster, whether up-regulated or down-regulated, are involved in the regulation of functionally associated genes [78]. Some miRNA clusters are typically up-regulated in CRC, including miR-106a/363, miR-106b/93/25, miR-17/92a-1, miR-181a-1/181b-1, miR-181a-2/181b-2, miR-181c/181d, miR-183/96/182, miR-191/425, miR-200c/141, miR-203a/203b, miR-222/221, miR-29b-1/29a, miR-301b/130b and miR-452/224 [79]. Others clusters are often downregulated in CRC including clusters like the miR-100/let-7a-2/miR-125b-1, miR-99a/let-7c, miR-99b/let-7e/miR-125a, miR-1-2/133a-1, miR-1-1/133a-2 and miR-206/133b, miR-192/194-2 and miR-215/194-1, miR-15a/16-1 and miR-15b/16-2, miR-143/145, miR-302b/302c/302a/302d/367, miR-497/195. Regarding the miR-23a/27a/24-2 cluster, there are conflicting data as it is usually reported as upregulated in CRC, whereas other studies demonstrated its downregulation with a consequent tumor suppressor role (Table 1). A thorough understanding of the transcriptional regulation of these clusters occurring in CRC could lead to a multi-target specific therapeutic approach [80]. Furthermore, all the aforementioned miRNAs can have a potential value for CRC diagnosis, prognosis and susceptibility [81]. For instance, the analysis of the expression levels of the circulating miR-17-3p, miR-92a, and miR-29a analyzed in liquid biopsy sample of individuals at risk for CRC has been proposed as a diagnostic strategy for the early detection of this tumor. Similarly, miR-20a, miR-21, miR-106a, miR-181b, and miR-203 were associated with poor survival [82], while increased levels of miR-155, miR-223, miR-31 and miR-26b were correlated with MSI-H status [83].

**Table 1:** Summary of the most frequently dysregulated miRNA clusters in CRC.

Cluster	Dysregulation	Function/Role	Related Information	miRNA	Target	Reference
**miR-143/145**	Downregulated	Tumor suppressors	Frequently downregulated in CRC due to hypermethylation of their CpG islands.	miR-145	*IRS1*	[84]
					*MUC1*	[85]
					*BRAF* and *CD44*	[86]
					*IGF1R*	[87]
					*KLF5*	[86]
					*MDM2*	[88]
					*MYC*	[89]
					*NRAS*	[90]
					*FSCN1*	[91]
					*CDCA3*	[92]
					*MAPK1*	[93]
					*SIP1*	[94]
					*catenin δ-1*	[95]
					*YES* and *STAT1*	[96]
					*PAK4*	[97]
					*ERG*	[98]
					*SOX2*	[99]
				miR-143	*BRAF* and *CD44*	[86]
					*MDM2*	[88]
					*MYO6*	[100]
					*KRAS*	[101]
					*MAPK7*	[102]
					*DNMT3A*	[103]
					*HKII*	[104]
					*MACC1*	[105]
					*BCL2*	[100]
**miR-1-2/133a-1, miR-1-1/133a-2, miR-206/133b**	Downregulated	Tumor suppressors	myo-miRNAs, muscle-specific miRNAs generally down-regulated in CRC cell lines and tissue samples.	miR-133a	*AFTPH*	[106]
					*FSCN1*	[107]
					*LASP1*	[108]
					*RFFL*	[109]
				miR-133b	*PTBP1*	[110]
					*MET*	[111]
					*CXCR4*	[112]
				miR-206	*NOTCH3*	[113]
					*MET*	[114]
				miR-1	*PTBP1*	[110]
					*NOTCH3*	[115]
					*SMAD3*	[116]
**miR-497/195**	Downregulated	Tumor suppressors	Expression of miR-497-5p and miR-195-5p is down-regulated in CRC. Increased expression of miR-497-5p or miR-195-5p is associated with decreased cell proliferation, migration and EMT.	miR-497	*IGF1-R*	[117]
					*FRA1*	[118]
					*IRS1*	[119]
					*KSR1*	[120]
				miR-195	*CDK8*	[121]
					*YAP1*	[122]
					*BCL2*	[123]
					*BCL2L2*	[124]
					*γ-catenin*	[125]
					*CCNE1*	[126]
					*AXIN2*	[127]
**miR-15a/16**–**1,miR-15b/16**–**2**	Downregulated	Tumor suppressors	Although more frequently miR-15/16 in CRC is detected downregulated, there are also studies documenting up-regulation of miR-15/16 expression. Similarly, better survival is generally correlated with high expression of miR-15/16; however, an association of worse survival with high expression of miR-15/16 has also been documented.	miR-15a	*AP4*	[128]
					*GPX4*	[129]
					*SIRT4*	[130]
					*KDM4B*	[131]
				miR-15b	*DCLK1*	[132]
					*NF-κB1* and *IKK-α*	[133]
					*ACOX1*	[134]
				miR-16	*AP4*	[128]
					*BIRC5*	[135]
					*HMGA2*	[136]
					*ALDH1A3*	[137]
					*COX2*	[138]
					*ITGA2*	[139]
**miR-192/194**–**2 and miR-215/194**–**1**	Downregulated	Tumor suppressors	Frequently downregulated in CRC, the reported functions of miR-192/194-2 and miR-215/194-1 clusters indicate their tumour-suppressive roles as cell cycle arrest and inhibition of cell adhesion are often observed after their overexpression.	miR-192	*CAV1*	[140]
					*EIF5A2*	[141]
				miR-194	*VAPA*	[142]
					*KLK10*	[143]
					*MAP4K4*	[144]
					*THBS1*	[145]
					*PDK1, AKT2* and *XIAP*	[146]
					*SSH2*	[147]
					*SOX5*	[148]
					*SIRT1*	[149]
				miR-215	*EREG* and *HOXB9*	[150]
					*Atg14*	[151]
					*CDX1*	[152]
**miR-183/96/182**	Upregulated	Oncogenes	Frequently downregulated in CRC, miR-183/96/182 cluster promote migration, invasion and metastasis.	miR-183	*ATG5*	[153]
					*FOXO1*	[154]
					*PFN2*	[155]
					*DNAJB4*	[156]
					*RCN2*	[157]
				miR-96	*TPM1*	[158]
					*CPA6*	[159]
					*AMPKα2*	[160]
					*TP53INP1, FOXO1, FOXO3a*	[161]
				miR-182	*NAMPT*	[162]
					*CFL1*	[163]
					*TIAM1*	[164]
					*DAB2IP*	[165]
					*SATB2*	[166]
					*ST6GALNAC2*	[167]
					*FoxF2*	[168]
					*TSP-1*	[169]
**miR-17/92a**	Upregulated	Oncogenes	miRNAs of miR-17/92a cluster can act as oncogenes and promote proliferation, angiogenesis and inhibit differentiation and apoptosis.	miR-17	*HSPB2*	[170]
					*MFN2*	[171]
					*BLNK*	[172]
					*RUNX3*	[173]
					*PLCD1*	[174]
					*CADM2*	[175]
					*VEGFA*	[176]
					*P130*	[177]
					*VIM*	[178]
					*SPOP*	[179]
					*PTEN*	[180]
					*RND3*	[181]
					*hCNT1*	[182]
				miR-18a	*ING4*	[183]
					*BTG3*	[184]
					*PIAS3*	[185]
					*TBPL1*	[186]
				miR-19a	*PTEN*	[187]
					*IREB2*	[188]
					*CLCA4*	[189]
					*THBS1*	[190]
					*KRAS*	[191]
					*FOXF2*	[192]
					*NPEPL1*	[193]
				miR-19b	*FBXW7*	[194]
					*PPP2R5E*	[195]
					*ACSL1, ACSL4,* and *SCD*	[196]
				miR-20a	*ATG5* and *FIP200*	[197]
					*CXCL8*	[198]
					*PDCD4*	[199]
					*MICA*	[200]
					*FOXJ2*	[201]
					*PTEN*	[202]
					*SMAD4*	[203]
				miR-92a	*DKK3*	[204]
					*SOCS3*	[205]
					*NF2*	[206]
					*KLF4*	[207]
**miR-200c/141**	Upregulated	Biomarkers	miRNA members of the miR-200c/141 cluster are found to be frequently upregulated in CRC at both tissue and circulating levels.	miR-200c	*VLDLR*	[208]
					*KIF14*	[209]
					*JNK2*	[210]
				miR-141	*SIP1*	[211]
					*EGFR*	[212]
					*ZEB1* and *ZEB2*	[213]
					*MAP4K4*	[214]
					*DLC1*	[215]
**miR-203a/203b**	Upregulated	Biomarkers	miR-203a/203b are generally overexpressed in CRC and are associated with poor prognosis.	miR-203a	*PTEN*	[216]
					*RNF6*	[217]
					*PDE4D*	[218]
					*THBS2*	[219]
					*CREB1*	[220]
				miR-203b	*BCL2L1*	[221]
**miR-222/221**	Upregulated	Circulating biomarkers	Both members of the miR-222/221 cluster are positively correlated with disease recurrence and are frequently detected upregulated in in the circulation of patients with CRC.	miR-222	*SPINT1*	[222]
					*CD4*	[223]
					*ADAM-17*	[224]
					*MIA3*	[225]
					*ATF3*	[226]
					*BRG1*	[227]
					*MST3*	[228]
				miR-221	*SPINT1*	[222]
					*CD4*	[223]
					*TP53INP1*	[229]
					*CDKN1C/p57*	[230]
					*SOCS3*	[231]
					*RECK*	[232]
**23a/27a/24**–**2**	conflicting data	conflicting data	Members of miR-23a/27a/24-2 cluster has been proposed to control the cell cycle, cell proliferation, cell death and cell differentiation.	miR-23a	*SEMA6D*	[233]
					*APAF1*	[234]
				miR-27a	*c-Met*	[235]
					*BTG2*	[236]
					*RMST*	[237]
				miR-24	*NRP1*	[238]
**miR-29b-1/29a**	Upregulated	Biomarkers	miR-29b-1/29a seems to have a biomarker value for risk, recurrence, metastasis and survival outcome of CRC.	miR-29b-1	*SMAD3*	[239]
				miR-29a	*RPS15A*	[240]
					*TNFAIP3*	[241]
					*KLF4*	[242]
**miR-301b/130b**	Upregulated	Oncogenes	Members of the miR-301b/130b cluster act as oncogenes by promoting cell growth and migration and may serve as biomarkers for the diagnosis of CRC.	miR-301b	*HOXB1*	[243]
				miR-130b	*CHD9*	[244]
					*integrin α5*	[245]

These and other studies allowed researchers to identify miRNAs potentially involved in the development and progression of tumors, including CRC (Table 1).

Notably, all these studies report data obtained on tissue or liquid biopsy samples or both, however, it is important to discriminate the reasons behind altered tissue and circulating expression levels of miRNAs. Altered expression of miRNAs in CRC tissues can be determined by intrinsic changes within cancer cells, including genomic alterations (e.g., amplifications, deletions), epigenetic dysregulation (e.g., DNA methylation, histone modifications), disrupted transcription factor activity, and impaired miRNA processing mechanisms. These changes often reflect the biology of the tumor itself, its interaction with the surrounding microenvironment, and the broader pathological transformation of the organ [246,247].

Conversely, changes in the circulating expression of miRNAs may have a more complex and multifactorial origin. They can be actively secreted by tumor cells via exosomes, microvesicles, or protein complexes, or passively released as a consequence of tumor cell death (apoptosis or necrosis). However, not all circulating miRNAs are directly tumor-derived. Some may represent a physiological systemic response to the presence of tumor, involving immune modulation, inflammation, or stress signaling [248]. Therefore, while tissue-derived miRNAs provide insights into the tumor's molecular profile, circulating miRNAs may serve as minimally invasive biomarkers that reflect both tumor biology and the host's systemic response. A comprehensive understanding of these distinct yet interconnected sources of miRNA alterations is essential for the development of robust biomarkers and targeted therapeutic strategies in colorectal cancer.

### 3.1 microRNAs in colorectal cancer development

In approximately 80 % of cases, the pathogenesis of CRC follows the adenoma-carcinoma sequence. In the vast majority of these cases, the development of CRC starts with an *APC* mutation responsible for chromosomal instability and the gradual accumulation of molecular and epigenetic changes. The remaining 15–20 % of CRC cases arise via alternative pathways, such as defective mismatch repair systems, CIMP hypermethylation, or *BRAF* activation. From a molecular point of view, the tumor suppressor genes *APC*, *TP53*, *PTEN*, *TGFβ*, *SMAD4*, the oncogenes *KRAS*, *BRAF*, *HER2*, and the tumor-modifying genes *COX2*, *PPAR* and *CHEK2* play an essential role in the development of CRC [249]. All these genes cause the activation of inflammatory signaling pathways and oncogenic signaling pathways. Besides activating mutations, these signaling pathways are also finely regulated by single miRNAs or by miRNA clusters/groups. If the expression of these miRNAs is altered, the proper functioning of these important signaling pathways may be impaired [250].

Another mechanism promoting CRC development is mediated by chronic inflammation due to inflammatory diseases, including colitis and inflammatory bowel diseases (IBDs). Colitis and chronic inflammation are responsible for immune cell infiltration, oxidative stress and the production of pro-inflammatory cytokines which induce genetic and signal transduction alterations associated with neoplastic transformation [[251], [252], [253]]. In this intricate scenario, different miRNAs regulating interleukin production, oxidative stress and p53 signaling were identified as associated with both IBDs and CR,C suggesting the epigenetic regulation of colitis-mediated carcinogenesis [251].

The miR-143/145 cluster is highly expressed in the colon and is typically reported to be downregulated in CRC and other cancers. Importantly, miR-143/145 cluster is not expressed in colon epithelial cells but in mesenchymal cells such as fibroblasts and smooth muscle cells. Through regulation of multiple targets, these miRNAs exert potent effects on cancer cell growth and tumorigenesis [254]. miR-145 plays a crucial role in cancer biology by directly targeting the pluripotency factors *OCT4*, *SOX2*, and *KLF4*. These factors are integral to the maintenance of stem cell pluripotency, which is also regulated by transcription factors like NANOG, SOX2, OCT4, KLF4, LIN28, and c-MYC. In this context, the loss of miR-145 impairs differentiation and leads to increased levels of *OCT4*, *SOX2*, and *KLF4* [255]. Furthermore, miR-145 is an inhibitor of the embryonic stem cell program, promoting cell differentiation and inhibiting the proliferation of SW48 cells harboring *KRAS* mutation [256].

Another mechanism responsible for the development of CRC is the inactivation of the *APC* gene. *APC* encodes a large scaffolding protein that is part of the AXIN destruction complex, which is required for phosphorylation and degradation of β-catenin. β-catenin is a key effector of Wnt signaling that interacts with the HMG-box DNA-binding factor TCF4 (TCF/L2) to drive transcription of target genes. If *APC* loses its function, β-catenin levels increase. Most mutations in *APC* generate premature stop codons that lead to the production of truncated proteins depleted of β-catenin binding sites. Consequently, β-catenin accumulates and stimulates the Wnt signaling pathway, leading to active transcription of target genes. In this scenario, miRNAs can modulate Wnt signaling through the repression of some of the components of this pathway. A study assessed the relationship between the downregulation of the miR-143/145 cluster and genetic aberrations in *APC*. In particular, it has been proposed that the downregulation of the miR-143/145 cluster often occurs before the osnet of *APC* gene aberrations. Thus, it may be considered an important epigenetic event in the early phase of CRC development [257]. The miR-143/145 cluster can also modulate the Ras-MAPK pathway; specifically, miR-145 targets *EGFR*, *RASA1*, *MEKK,* and *RREB1*, while miR-143 targets *KRAS*, *ERK1/2*, and *ELK1*. Furthermore, the miR-143/145 proximal promoter is negatively regulated by the K-Ras-RREB1 feedback loop. Specifically, *RREB1* is activated by the MAPK pathway and negatively represses the miR-143/145 promoter through the interaction with two Ras-responsive elements (RREs) [254,258]. Other recognized targets of miR-145 are insulin receptor substrate 1 [84], Src-related tyrosine kinase *YES* [96], *c-MYC* and *ERK5* [259], *catenin δ-1* [95], *PXN* [260], *FSCN1* [91], *MUC1* [85]. Many studies have also identified several targets for miR-143, such as *MDM2* [88], *HKII* [104], *DNMT3A* [103], *MAPK7* [102], *KRAS* [101], *BRAF* and *CD44* [86]. All these factors, when dysregulated, promote CRC development by increasing cell cycle progression, cell proliferation, cell metabolism, cell survival, immune evasion, and metastasis formation.

The *miR-23b/27b/24* cluster has two paralogs in humans, the *miR-23b/27b/24-1* cluster, which is encoded within an intron on the *C9orf3* gene located on chromosome 9, and *miR-23a/27a/24-2* located on chromosome 19. The miR-23b/27b/24 cluster seems to have a role in cell migration by targeting *FOXP2* through *miR-23b* and *miR-27b* [261]. Although this cluster is generally found to be upregulated, several studies have reported a downregulation and a tumor suppressor role of its members in CRC. miR-27b acts as a tumor suppressor miRNA by targeting *ARFGEF1* and the paxillin/c-Src circuit at focal adhesions [262]. miR-23b has pleiotropic functions; thus if dysregulated, it can lead to a variety of diseases, including cancer. In CRC, the downregulation of *miR-23b* modulates the expression levels of its target *PDE7A*, which is involved in the development of this tumor [263]. miR-27a plays a critical role in colon tumorigenesis, possibly influencing the anti-tumor immune response. Specifically, miR-27a modulates MHC surface exposure by targeting calreticulin, a highly conserved chaperone protein, important for the assembly and expression on the cell surface of MHC class I molecules and thus for the recognition of the presented tumor-associated antigen by CD8 T-cells [264].

The miR-10a/b, miR-99a/b, miR-100 and miR-125a/b, constituting the miR-10 family, possess tumor-suppressive properties. miR-100 targets *RAP1B* and modulates CRC cell growth and invasion phenotype [265]. miR-125b targets *TP53* and other regulators of apoptosis, including *PUMA*, *BAK1* and cyclin C, thus regulating cell cycle transition [266]. Moreover, miR-99, miR-100, and miR-125 genomic loci are physically clustered with the loci encoding for the let-7 miRNA family. Therefore, chromosomal deletions or transcriptional silencing of these genomic regions may influence both miR-10 and miR-let-7 families, although no validations of these hypotheses have been documented yet. Notably, let-7 family members play an important tumor-suppressor role due to their anti-proliferative function and pro-differentiation effects. LIN28A and LIN28B are specific and strong inhibitors of let-7 members by interfering with the biogenesis of the whole let-7 family [267]. Indeed, LIN28B is found overexpressed in several tumor types, including CRC, where it promotes colon cell malignant transformation through the suppression of let-7 [268].

The miR-34a, miR-34b and miR-34c family members regulate the expression of genes involved in the cell cycle, cell growth, DNA damage repair and apoptosis. miR-34a and miR-34b/c are transcribed from two different loci, both direct transcriptional targets of the tumor suppressor *TP53* [269,270]. In turn, miR-34 directly represses *MDM4* (*HDM4* in humans), which encodes a RING-finger protein that binds p53 and blocks its ability to activate target genes. Thus, miR-34a may promote tumorigenesis, especially in the case of p53 haploinsufficiency [271]. A study performed by Gao J et al. on a cohort of 268 CRC patients showed that miR-34a-5p inhibits CRC metastasis by repressing cell growth, migration and invasion, inducing cell apoptosis and cell cycle arrest in a p53-dependent manner [272]. p53 transactivates miR-34, which represses the transcriptional activity TCF/LEF complexes by targeting genes encoding elements of the Wnt pathway. Thus, in CRC, loss of p53 or miR-34 promotes neoplastic progression, enhancing the Wnt signaling [273]. The expression of miR-34 may also depend on its methylation status. For instance, in FFPE colon cancer samples compared to normal colon mucosa, miR-34 was downregulated due to promoter hypermethylation [274]. Besides *TP53*, *MYC* can also promote the expression of miR-34. MK5 indirectly regulates *MYC* translation by activating the expression of miR-34b and miR-34c, which in turn bind the 3′UTR of *MYC*. Specifically, MK5 phosphorylates FoxO3a, thereby promoting its nuclear localization, inducing miR-34b/c expression and the inhibition of cancer cell proliferation [275]. The miR-34 family also plays a role in the regulation of tumor-initiating cancer stem cells (TICs). In CRC, TICs generally present intrinsic drug resistance mechanisms leading to chemotherapeutic failure. Such drug resistance mechanisms seem to be associated with miR-34a and miR-146 dysregulation [276]. miR-34a is a cell fate determinant in early-dividing colon cancer stem cells (CCSCs). Specifically, miR-34a targets Notch1 mRNA to generate a net threshold response in which a bimodal Notch signal specifies the choice between self-renewal and differentiation enabling cells to distinctly choose between maintaining a stem-like state or committing to differentiation [277]. These data suggest that miRNAs can indirectly promote asymmetric division, but it remains unclear whether and how miRNAs and proteins drive the cell fate. Another study showed that miR-34a targets Numb in early CCSCs and inhibits asymmetric division in cooperation with miR-146 [278]. In this scenario, it is known that the correct number of stem cells for self-renewal is maintained through asymmetric cell division (ACD). In cancer cells, the deregulation of ACD causes an alteration of the stem cell pool and promotes tumor growth. The EMT inducer Snail is responsible for the switch from ACD to symmetrical cell division (SCD) in CRC. Specifically, Snail induces the expression of miR-146a via the β-catenin-TCF4 complex. In turn, miR-146a targets Numb to stabilize β-catenin, which forms a feedback loop to maintain Wnt activity and directs SCD [279].

Another key relevant miRNA family is that of the miR-17/92 cluster, whose miRNAs actively cooperate with several oncogenic miRNAs, including miR-21-5p, miR-31, miR-135b and miR-145. All these miRNAs were investigated in clinically diagnosed early-stage CRC (24 colonic polyps containing early-stage adenocarcinoma). In particular, miR-17 showed increased expression in the transition zone from normal to adenomatous tissue, while miR-21-5p expression increased in the tumor-associated stroma, with an even more evident increase from adenoma to adenocarcinoma; in contrast, miR-145 expression decreased gradually during the normal-adenoma-adenocarcinoma progression. Therefore, these miRNAs may play a role in CRC development [280].

The miR-17/92 cluster, which includes miR-17, miR-18a, miR-19a, miR-20a, miR-19b, and miR-92a, is commonly upregulated in both hematological malignancies and solid tumors, including CRC [281,282]. Its overexpression is often associated with c-Myc activation and copy number gain of its locus on chromosome 13q31 [283]. Functionally, the members of the miR-17/92 family promote cell proliferation and angiogenesis, while inhibiting differentiation and apoptosis by modulating key oncogenic signaling pathways, such as JAK/STAT, PI3K/AKT/mTOR, and PTEN [280,284]. The expression of the miR-17/92 cluster is also modulated by the APC-β-catenin pathway; specifically, activated β-catenin resulting from APC loss can bind to and activate the miR-17/92 promoter region [285]. The expression of miR-18a correlates with *APC* mutations and is highly expressed in colon cancer [68]. In CRC, miR-20 influences the activation of the cyclin-dependent kinase inhibitor 1A/p21 (*CDKN1A/p21*), which negatively regulates *TGFβ*, thus preventing its antiproliferative effect [286]. miR-17/92 cluster is also associated with invasion, metastasis and decreased survival. Of the six members of the miR-17/92 cluster, miR-19a and miR-19b have been described as key promoters of cancer development and cancer cell proliferation. Even belonging to the miR-17/92 cluster, the miR-18a plays a conflicting role in CRC since it was found downregulated in CRC, suggesting that this miRNA may have tumor-suppressive effects compared to the other members that are often found overexpressed and associated with CRC cell proliferation [287]. Humphreys KJ et al., suggested that individual miR-17/92 cluster members have opposite effects on CRC cell proliferation. Specifically, miR-19a and miR-19b were primarily responsible for increased cell proliferation, while miR-18a showed the opposite effect by silencing the transcription of genes involved in cell proliferation, such as *NEDD9* and *CDK19* [288]. Thus, high miR-17/92 cluster activity without an increase in miR-18a can promote CRC progression. Indeed, while other members of the miR-17/92 cluster activate the PI3K pathway, thereby promoting cell growth, miR-18a can suppress growth by targeting *CDC42* and *CCND1* [287]. In addition, several post-transcriptional regulatory mechanisms influence the abundance of specific members of the miR-17/92 cluster. For instance, it was observed that miR-18a is the only member of the miR-17/92 cluster that requires the RNA-binding protein hnRNPA1 for its processing [289]. Furthermore, pri-miR-17-92 has a compact globular tertiary structure, which makes difficult the maturation of miRNAs [290].

Besides its role within the miR-17/92 cluster, miR-92a is also a member of a conserved miRNA family including miR-92a-1, miR-92a-2, miR-363 and miR-25. miR-92a is overexpressed in several tumors and its upregulation was associated with poor long-term prognosis in CRC [291]. In CRC, miR-92a exerts its tumorigenic role by influencing several mechanisms that lead to the downregulation of tumor suppressor and apoptotic genes and the upregulation of genes involved in cell proliferation [64]. Yamada N. et al., suggest that at the intracellular level, miR-92a targets *DKK3*, while when secreted through MVs this miRNA promotes angiogenesis [292]. It was also demonstrated that the expression levels of miR-92a are positively regulated by the pro-inflammatory IL-6/STAT3 pathway. As a result, miR-92a targets *KLF4*, *GSK3β* and *DKK3* involved in the negative regulation of Wnt/β-catenin signaling [293]. In addition, miR-92a plays a crucial role in the regulation of apoptosis by targeting the anti-apoptotic molecule BCL-2-interacting mediator of cell death (*BIM*) [294].

Moreover, miR-17 belongs also to the miR-17 family consisting of miR-17, miR-18a/b, miR-20a/b, miR-93, and miR-106a/b. miR-17-5p is an oncogenic miRNA that regulates cancer development and progression by targeting P130 (RB transcriptional co-repressor like 2, *RBL2*) and subsequently activating the Wnt/β-catenin pathway [177]. Transfection of CRC cells with a miR-17 inhibitor reduced the proliferation of cancer cells by inducing G0/G1 arrest via *RND3* targeting [181]. Ataxia telangiectasia mutated (*ATM*) gene encodes a key enzyme involved in DNA damage repair. *ATM* transcript is targeted by miR-18a that, when overexpressed in CRC, affects DNA damage repair [295,296]. miR-20a affects the cellular response to TGF-β and favors G1/S transition, promoting cell cycle progression [297]. miR-106b appears to have functions in the EMT of CRC. Indeed, miR-106b downregulation induces cytoskeletal reorganization and increases the expression of Rho GTPases (*RAC1* and *CDC42*) and *TIAM1*. *TGF-β1* can downregulate miR-106b and in turn, miR-106b also influences *TGF-β1* expression, establishing a negative feedback loop that regulates the expression of *PRRX1*, a direct target of miR-106b [298].

The miR-135a/b family is often upregulated in CRC and targets *APC,* thus suppressing its expression and inducing the downstream activation of the Wnt pathway [299]. Other miR-135a/b targets associated with the Wnt signaling pathway are the secreted frizzled-related protein 4 (*SFRP4*), which binds and represses extracellular Wnt proteins [300] and *ZNRF3*, which is involved in the negative regulation of the Wnt pathway [301]. Valeri N et al., demonstrated that the overexpression of miR-135b is associated with *APC* loss, the deregulation of the PTEN/PI3K pathway and the overexpression of *SRC*. The upregulation of miR-135b also promotes malignant transformation and tumor progression, especially in sporadic and inflammatory bowel disease-associated human CRC. The overexpression of this miRNA also correlates with tumor stage and poor patients’ prognosis [302].

Besides those already mentioned families, several other miRNAs involved in the regulation of Wnt/β-catenin signaling have been identified. In particular, miR-552 is able to regulate the Wnt/β-catenin signaling pathway by targeting the cell fate-determining factor Dachshund family transcription factor 1 (*DACH1*) [303]. C-MYC can stimulate the expression of miR-552 by binding the miR-552 promoter. In turn, miR-522 targets *TP53* exerting its oncogenic properties [304]. miR-590-3p targets *WIF1* which inhibits WNT and DKK1 and in turn the LRP6 co-receptor inhibiting the β-catenin-dependent Wnt signaling [305]. miR-425-5p may promote tumorigenesis and metastasis by activating catenin-δ1 (*CTNND1*) mediated β-catenin pathway [306]. miR-29b, miR-29c and miR-93 are other inhibitors of Wnt ligands or β-catenin-associated factors. miR-29b targets *BCL9L*, a co-activator of β-catenin [307], miR-29c targets *GNA13* and *PTP4A* [308]; miR-93 targets *SMAD7*, which promotes nuclear accumulation of β-catenin [309]; miRNA-29a targets the phosphoinositide 3-kinase, phosphorylated (p)-protein kinase B (*AKT*), p-glycogen synthase kinase 3β (*GSK3β*) [310].

Finally, one of the most widely studied oncomiRs involved in CRC pathogenesis is miR-21-5p, which is responsible for the regulation of multiple tumor-promoting mechanisms. A recent study detected high levels of miR-21-5p in CRC-derived exosomes. The authors demonstrated that the treatment of colon cells with isolated CRC-derived exosomes or miR-21-5p mimic leads to increased expression of genes involved in cell proliferation, invasion and extracellular matrix formation, including *PDCD4*, *TPM1*, and *PTEN* [311]. Particularly, PDCD4 is a pro-inflammatory factor that is activated by apoptotic stimuli and inhibits tumor proliferation by modulating NF-κB activity. In the case of low miR-21-5p expression, inflammatory infiltration decreased and fewer tumor-associated inflammatory cytokines, such as TNF-α, IL-6, IL-17A and IL-21, were produced. Thus, miR-21-5p seems to promote the development of colon cancer by promoting inflammation [312]. Furthermore, another study observed that miR-21-5p expression increased during the transition from precancerous colorectal adenoma to advanced carcinoma. In addition, the expression patterns of miR-21-5p and its target *PDCD4* were mutually exclusive [313]. miR-21-5p may also potentiate TCF4/β-catenin-mediated transcriptional activation [314,315]. Lin PL et al., analyzed the APC mutation from 165 CRC samples and found that miR-21-5p was associated with β-catenin phosphorylation at Ser552 via the PTEN/AKT axis and played a critical role in β-catenin nuclear translocation in APC-mutated cells, but not in APC-wild-type cells [314].

All these studies indicate that CRC-associated oncogenic and tumor-suppressive signaling pathways and inflammatory pathways are finely regulated by specific miRNAs, such as miR-145, miR-34, and the miR-17/92 cluster, which play essential roles in modulating cell differentiation, proliferation, apoptosis, and immune response. Dysregulation of these miRNAs fosters CRC progression through mechanisms like stem cell pluripotency, Wnt signaling, and EMT, underscoring their potential as therapeutic targets (Table 2).

**Table 2:** miRNA clusters and their targets involved in CRC development.

miRNA/Cluster	Key Targets	Pathways Affected	Role in CRC	References
**miR-145**	*OCT4*, *SOX2*, *KLF4*, *EGFR*, *RASA1*, *MEKK*, *RREB1*	Wnt, Ras-MAPK, Pluripotency factors	Tumor suppressor, downregulated in early CRC, promotes cell differentiation	[[255], [256], [257]]
**miR-143**	*KRAS*, *ERK1/2*, *ELK1*, *MDM2*, *HKII*, *DNMT3A*	Ras-MAPK, Cell cycle, Metabolism	Tumor suppressor, modulates KRAS pathway, downregulated early in CRC	[257][88,101,104]
**miR-23b/27b/24 Cluster**	*FOXP2*, *ARFGEF1*, *PDE7A*	Cell migration, c-Src circuit, Immune response	Tumor suppressor, downregulation affects cell migration, immune response modulation	[[261], [262], [263]]
**miR-10 Family (miR-10a/b, miR-99a/b, miR-100, miR-125a/b)**	*RAP1B*, *p53*, *PUMA*, *BAK1*, *Cyclin C*	Apoptosis, Cell cycle, Invasion	Tumor suppressor, regulates apoptosis and cell growth, downregulation in CRC	[265,266]
**miR-let-7**	*-*	Proliferation, Differentiation	Tumor suppressor, let-7 regulates differentiation, suppressed by LIN28 A/B	[268]
**miR-34 Family (miR-34a/b/c)**	*MDM4*, *β-catenin*, *Numb*	p53, Wnt, DNA damage response	Tumor suppressor, regulates cell cycle and apoptosis, loss of miR-34 linked to p53 deficiency	[269,271,272]
**miR-17**–**92 Cluster (miR-17, miR-18a, miR-19a/b, miR-20a, miR-92a)**	*NEDD9*, *CDK19*, *PTEN*, *Cyclin D1*	PI3K/AKT/mTOR, JAK/STAT, Cell proliferation, Apoptosis	Oncogenic, promotes proliferation, inhibits apoptosis, miR-18a tumor-suppressive, others oncogenic	[281,282,285,286]
**miR-92a**	*DKK3*, *KLF4*, *GSK3β*, *BCL-2*, *BIM*	Wnt/β-catenin, Apoptosis	Oncogenic, overexpression leads to poor prognosis, targets tumor suppressor genes	[291,293,294]
**miR-135a/b**	*APC*, *SFRP4*, *ZNRF3*	Wnt, PTEN/PI3K	Oncogenic, suppresses APC and activates Wnt signaling	[299,301,302]
**miR-522**	*TP53*, *DACH1*	Wnt/β-catenin	Oncogenic, stimulated by c-Myc, regulates Wnt pathway via TP53 targeting	[303,304]
**miR-590-3p**	*WIF1*, *DKK1*	Wnt	Inhibits Wnt/β-catenin signaling by targeting WIF1 and DKK1	[305]
**miR-425-5p**	*CTNND1* (*catenin δ-1*)	β-catenin pathway	Promotes tumorigenesis and metastasis	[306]
**miR-29 Family (miR-29a/b/c)**	*BCL9L*, *GNA13*, *PTP4A*	Wnt/β-catenin	Regulates β-catenin co-activators	[307,308]
**miR-93**	*SMAD7*	Wnt/β-catenin	Inhibits SMAD7 and promotes β-catenin accumulation	[309]
**miR-21**	*PDCD4*, *TPM1*, *PTEN*	NF-κB, Inflammation, ECM formation	Oncogenic, promotes tumorigenesis by inducing inflammation, upregulated in CRC exosomes	[[311], [312], [313]]

In this intricate genetic and epigenetic scenario, other ncRNAs have been found to influence miRNA expression, adding another layer to CRC pathogenesis. Indeed, miRNA dysregulation may be due to aberrant transcriptional activity, a change in epigenetics, altered miRNA biogenesis, as well as sponging of lncRNAs. For instance, miR-200a and miR-138, known to attenuate EMT, are modulated by the H19 lncRNA that is upregulated in CRC tissues [316]. Another lncRNA-miRNA interaction found in CRC is between LINC00152 and miR-139-5p, which results in increased cell proliferation, promotion of metastasis, and confers resistance to 5-FU [317]. NEAT1 lncRNA is up-regulated in CRC tissues and correlates with poor overall and disease-free survival. NEAT1, functioning as a ceRNA, modulates miRNA-34a expression, resulting in the repression of the miR-34a/SIRT1 axis and in the activation of the Wnt/β-catenin signaling pathway [318]. XIST is another lncRNA that negatively modulates miR-34a expression, leading to an increase of its target WNT1 [319]. ZEB1-AS1 lncRNA is significantly upregulated in CRC and promotes CRC cell proliferation, repressing apoptosis via the downregulation of miR-181a-5p and positively regulating the Wnt/β-catenin signaling [320]. The downregulation of miR-181a-5p can also be mediated by CRNDE lncRNA sponging, which results in the inhibition of cell proliferation and the reduction of chemoresistance [321]. The lncRNA MIR4435-2HG increases tumor growth and metastasis formation by sponging miR-206 that regulates the Yes-associated protein 1 (*YAP1*) transcription factor, a major effector and downstream regulator of the Hippo pathway [322]. *YAP1* expression is also regulated by miR-139-5p, which in turn is regulated by the overexpressed oncogenic lncRNA RP11-757G1.5 [323]. The LINC00689 lncRNA can target miR-31-5p. In CRC, LINC00689 is downregulated, while miR-31-5p is upregulated. The target of miR-31-5p, Large Tumour Suppressor Kinase 2 (*LATS2*), phosphorylates YAP1, which regulates genes involved in cell proliferation, death, and migration. Furthermore, the activation of YAP1 could stimulate the activity of other transcription factors such as SMAD, trigger EMT and thus increase metastasis and invasiveness of cancer cells [324].

### 3.2 microRNAs in colorectal cancer progression

Besides their key role in CRC development, miRNAs also influence CRC progression and aggressiveness. Indeed, by targeting genes involved in EMT, apoptosis, cell growth and proliferation, miRNAs can promote angiogenesis, metastasis and tumor progression [325].

miR-155 regulates a variety of cellular functions, including EMT. The expression level of miR-155 is higher in primary CRC tissue than in adjacent normal mucosa. miR-155 has been shown to increase the migratory and invasive capacity of SW480 inducing claudin-1 expression [326]. Through RNA sequencing, another study revealed high levels of miR-146a-5p and miR-155-5p in CRC cells overexpressing the C-X-C motif chemokine receptor 7 (*CXCR7*). Specifically, CXCR7 binds the C-X-C motif chemokine ligand 12 (CXCL12), favoring the formation of CRC metastasis. In this process, CAFs are also involved in tumor progression through the secretion of both miR-146a-5p and miR-155-5p via exosome trafficking. In particular, CAFs may take up these miRNAs promoted by the JAK2-STAT3/NF-κB signaling. With a positive feedback loop, CAF-produced miR-146a-5p and miR-155-5p target the suppressor of cytokine signaling 1 (*SOCS1*) and the zinc finger and BTB domain containing 2 (*ZBTB2*), promoting the production of inflammatory cytokines, including IL-6, TNF-α, TGF-β and CXCL12 favoring tumor progression [327].

Tumor-associated macrophages (TAMs) can also promote CRC initiation and progression by influencing miRNA expression. Since exosomes derived from M2 macrophages (MDE) have high levels of miR-21-5p and miR-155-5p, they may contribute to migration and invasion in CRC [328]. Moreover, the tumor-promoting role of miR-155-5p was also observed in FAP patients *vs* non-FAP controls, where a significant downregulation of miR-155-5p expression was found in FAP patients and APC and β-catenin mutant colorectal cancer cell lines. Furthermore, miR-155-5p can regulate WNT/β-catenin signaling by targeting both *AXIN1* and *TCF4* [329].

miR-34 also seems to play a role in CRC progression. The expression of miR-34 in CRC is significantly downregulated. In SW480 cells, miR-34a attenuates migration and invasion by targeting Notch1 and Jagged1, suggesting a key role in suppressing CRC metastasis [330]. The ectopic expression of miR-34a in HCT-116 and RKO colon cancer cell lines caused complete suppression of cell proliferation and induced senescence-like phenotypes through the modulation of the E2F signaling pathway [331]. Other studies confirmed the role of miR-34 in the suppression of EMT. Specifically, *ZNF281* is one of the putative targets of miR-34. Noteworthy, *SNAIL* induces EMT by activating *ZNF281* transcription and repressing miR-34a/b/c, which cannot inhibit *ZNF2*81 mRNA. Besides its role in EMT, *ZNF281* overexpression also induces the stemness markers LGR5 and CD133 [332]. As described before, p53 transcriptionally activates miR-34a and, in turn, miR-34a downregulates the expression of silent information regulator 1 (*SIRT1*). By suppressing miR-34, *SIRT1* promotes apoptosis in WT human colon cancer cells but not in those with deficient p53 [333]. miR-34 also plays a key role in inflammation as demonstrated by the active loop involving IL-6R/STAT3/miR-34a, which is required for CRC EMT, invasion and metastasis. This axis is also associated with lymph node and distant metastasis in CRC patients [334].

Communication between tumor cells and blood capillaries plays an important role in tumor growth, invasion, and spreading. A coculture assay showed that SW480 cells form functional gap junctions composed of connexin-43 (*CX43*) with human microvascular endothelial cells (HMECs). By overexpressing miR-145-5p in HMECs, the level of miR-145 also increases dramatically in SW480. In SW480 cells, miR-145 regulates the expression of CX43 and inhibits its pro-angiogenic capabilities. However, although miR-145 is transferred from SW480 cells to HMECs, the exact mechanisms underlying this transfer remain unclear. Notably, this transfer does not occur in non-contact co-cultures, thus excluding the involvement of soluble exosomes [335].

CRC progression and metastasis are prompted by constitutive and epigenetic RAS activation. In pancreatic cancer, it was demonstrated that RAS signaling leads to the repression of the miR-143/145 cluster. The downregulation of this cluster may be due to the repression of the cluster promoter operated by *RREB1*. Both *KRAS* and *RREB1* are targets of miR-143/145, suggesting a feed-forward mechanism that enhances RAS signaling [336]. Certain circRNAs bind miRNAs and sequester them by inhibiting their functions. Hsa_circ_001569 promotes the proliferation and invasion of CRC cells by sponging miR-145 and induces the upregulation of miR-145 targets like *E2F5*, *BAG4*, and *FMNL2* [337].

miR-20a and miR-92a belong to the miR-17/92 cluster and are overexpressed in CRC. miR-20a expression seems to correlate with lymph node metastasis and distant metastasis. Transfection of SW480 CRC cells with miR-20a promoted migration and invasion and induced EMT in CRC cells partly through the suppression of *SMAD4* expression [203,338]. In SW480 cells, miR-92a induced EMT and regulated cell growth, migration and invasion via PTEN [339]. Moreover, miR-92a can promote CRC invasion and migration by targeting *RECK* [291].

The downregulated miR-124 has several biological functions and is involved in cell proliferation, autophagy and neuronal differentiation. miR-124 is abnormally expressed in inflammatory diseases and immune disorders by acting as an inhibitor of the inflammatory response [340]. A study analyzed the effects of methylation, overexpression and downregulation of miR-124 revealing how miR-124 suppresses CRC proliferation, migration and invasion by targeting *DNMT3B* [341]. Furthermore, miR-124 can modulate autophagy and apoptosis in CRC cells by inhibiting *STAT3* [342,343] and the polypyrimidine tract-binding protein 1 (*PTBP1*) [344].

The miR-200 family, consisting of miR-200a/b/c, miR-141 and miR-429, located in two gene clusters, is often reported to be associated with epithelial differentiation and repression of EMT [345]. This miRNA family is also found downregulated in CRC due to promoter methylation [346]. As regards each family member, miR-200 was found to directly target the mRNA of the pro-mesenchymal transcription factors *ZEB1*, *ZEB2* and *PRRX1* [347]. Moreover, miR-200 suppresses EMT and metastasis and targets *PD-L1*, acting as a tumor suppressor miRNA. However, miR-200 is transcriptionally repressed by ZEB1, an activator of EMT, inducing the overexpression of *PD-L1* and leading to immunosuppression of CD8(+) T-cells and metastasis [348]. As an example of the role of the miR-200 family in the onset of metastasis, miR-200c and miR-141 were found to be overexpressed in liver metastases compared to primary CRC tumors [349]. In agreement with these results, it was shown that serum levels of miR-200c are also high in patients with CRC metastases [350]. miR-141 by targeting *SIP1* affects migration and invasion of CRC cells [211]. miR-141-3p negatively regulates proliferation, migration and invasion and sensitizes CRC cells to cetuximab through suppression of *EGFR*, thus serving as a potential predictive biomarker for response to cetuximab [212]. These miRNAs are also modulated by other ncRNAs, including H19 lncRNA, which activates the β-catenin pathway by sponging miR-141. Furthermore, H19 is highly expressed in CRC samples and has been associated with colorectal cancer stem cell (CSC). H19 has also been detected in CAF-derived exosomes, which in turn promote CSC stemness and chemoresistance of CRC cells [351].

The role of CAFs in the secretion of metastasis-inducing miRNAs was also observed for miR-31. Specifically, the expression of miR-31 was found to be increased in colorectal CAFs compared to normal colorectal fibroblasts (NFs). Overexpression of miR-31 in CAFs represses the expression of the autophagy-related genes *BECN1*, *ATG*, *DRAM*, and *LC3*, with negative effects on cell proliferation, invasion and apoptosis, and positive effects on CRC cells radiosensitivity [352].

MiR-200c, miR-17, and miR-192 were identified as major miRNAs involved in the regulation of genes crucial for extracellular matrix remodeling. Accordingly, expression of these miRNAs in human colon fibroblasts co-cultured with colon cancer cells significantly reduced cancer cell invasion [353]. A recent study compared the expression of selected miRNAs and EMT markers in biopsy samples from patients (n = 45) with primary CRC or metastatic CRC. The study indicated miR-17, miR-19b, miR-106a and miR-9 and the EMT-specific markers *MMP2* and *VEGFA* as biomarkers with potential diagnostic, predictive and prognostic values in CRC progression and metastasis [354]. In addition, miR-106a was found to be highly expressed in metastatic CRC cells and seems to promote migration and invasion of tumor cells by targeting transforming growth factor receptor β (*TGFBR2*) [355].

RAN binding protein 1 (*RANBP1*) expression has been strongly associated with TNM stages and poor prognosis. RANBP1 could affect the nucleocytoplasmic transport of the pre-miRNAs of miR-18a, miR-183 and miR-106 and promotes *YAP* expression by influencing the Hippo pathway. YAP in turn functions as a transcriptional cofactor together with TEAD4 to activate *RANBP1* transcription [356].

A study evaluated the role of miR-181a in tumor angiogenesis. miR-181a targets SRC kinase signaling inhibitor 1 (*SRCIN1*), resulting in the activation of SRC and the subsequent secretion of VEGF, leading to increased angiogenesis [357]. Moreover, the expression of miR-181a is higher in CRC with liver metastases; indeed, high levels of miR-181a correlate with advanced-stage, distant metastases and serve as an independent prognostic factor of poor overall survival. The overexpression of miR-181a in CRC cells promotes cell motility and invasion partly due to the inhibition of expression of its target *WIF1* [358].

The expression of miR-15 and miR-16 is regulated by the promoter of their host gene *SMC4* [359]. miRNA 16-1 is frequently deleted or downregulated in several tumors, including CRC, where it plays a role in EMT, contributing to the capacity of CRC cells to metastasize [128]. Most of the targets of miR-15a-5p and miR-16-5p in CRC are genes involved in EMT regulation, such as *CCNB1* [360] or transcription factor *AP4* [128]. AP4 is a helix-loop-helix transcription factor encoded by c-MYC which is upregulated in CRC. A study identified hundreds of induced and repressed AP4 target genes. Other gene targeted by AP4 are the stemness markers *LGR5* and *CD44* as well as genes involved in EMT, such as *SNAIL*, *E-cadherin/CDH1*, *OCLN*, *VIM*, *FN1* and *claudins 1*, *4* and *7*. Hence, AP4 promotes EMT and increases the migration and invasion of CRC cells [361]. In clinical CRC samples, miR-15a levels are inversely correlated with AP4 protein levels, which in turn correlate with distant metastasis and poor survival [128].

miR-206 is frequently downregulated in many human malignancies, including CRC. miR-206 suppresses CRC cell proliferation by arresting CRC cells in the G1/G0 phase, accelerates apoptosis, and inhibits cell invasion by targeting *FMNL2* and *c-MET* [362]. In HCT116 and Caco-2 cells treated with prostaglandin E2 (*PGE2*), the expression of miR-206 decreases while the expression of its target *TM4SF1* increases, resulting in cell proliferation and repression of apoptosis [363]. *NOTCH3* is an established target of miR-206, frequently expressed in human CRC samples and involved in CRC cell modulation and tumorigenic potential. Transient transfection of miR-206 mimic into SW480 and SW620 cells results in the inhibition of cell proliferation, cell cycle blockade and activation of apoptosis through downregulation of *NOTCH3* and potential indirect inhibition of other signaling pathways involving *CDH2* and *MMP9* [113]. miR-206, together with miR-1 and miR-133a/b, belongs to the group of myo-miRNAs that are muscle-specific miRNAs [364]. Besides miR-206, another myo-miRNA generally down-regulated in CRC cell lines and tissue samples is miR-133a. Ectopic expression of miR-133a inhibited cell proliferation and migration. Stable overexpression of miR-133a was sufficient to suppress tumor growth and intrahepatic and pulmonary metastasis *in vivo* [108]. It was observed that in CRC, overexpression of *CXCR4* promotes EMT and the infiltration of myeloid-derived suppressor cells (MDSCs) and macrophages into colonic tissue, accelerating APC mutation-associated colitis and CRC progression. In addition, it was observed that miR-133a-3p significantly decreased after XIST sponging, determining an increase of the target RhoA, which is involved in cytoskeletal reorganization and cell motility in HCT116 cells [365].

The expression levels of miR-320a in CRC cell lines and tumor tissues were found to be frequently downregulated. The restoration of miR-320a inhibited CRC cell proliferation and repressed its direct target β-catenin [366]. Similarly, a lentiviral-mediated re-expression of miR-320c inhibits the growth and migration of HCT116 and sensitizes CRC cells to 5-FU [367].

*TET1*, downregulated in CRC, is a miR-21-5p target that acts as a tumor suppressor and inhibits cell growth [368]. Moreover, miR-21-5p can enhance cell migration, intravasation, and metastasis by targeting programmed cell death 4 (*PDCD4*) [369].

Several immune factors contribute to the progression of CRC. For instance, myeloid-derived granulocyte suppressor cells (G-MDSCs) increase cancer growth. CRC tissues have been found to contain G-MDSC cells that secrete exosomes containing miR-166-5p. These exosomes accelerate cancer progression by promoting cell proliferation. miR-166-5p by targeting integral membrane protein 2B (*ITM2B*), which in turn activates the PI3K/Akt signaling pathway, promotes cell proliferation in CRC [370].

Overexpression of miR-195-5p in DLD1 and HCT116 cells represses cell growth, colony formation, invasion and migration by suppressing the Hippo-YAP pathway by targeting *YAP* [122].

The expression of miR-203 was quantified in primary CRC (pCRC) and corresponding liver metastasis (LM) and serum samples from CRC patients. The expression of miR-203 was significantly upregulated in LM compared to the corresponding pCRC tissues. Serum levels of miR-203 were elevated in a stage-dependent manner and high miR-203 expression was associated with poor survival in CRC patients in both patient cohorts [371].

miR-23b also appears to play a role in metastasis by interacting with *BTBD7* [372]. miR-23a is overexpressed in CRC cell lines and tissues and regulates *PDK4* expression by targeting its mRNA. PDK4 negatively regulates CRC proliferation through suppression of PDH activity. Accordingly, up-regulation of miR-23a promotes CRC cell proliferation by directly repressing *PDK4* [373]. A study showed that miR-23a was significantly elevated in MSI CRC cells and tissues compared to CRC cells and tissues with stable microsatellite status (MSS). Ectopic expression of miR-23a increased the viability and survival of CRC MSS cells, while the downregulation of miR-23a reduced viability and promoted cell apoptosis in CRC MSI cells treated with 5-FU. In these models, *ABCF1* was found as a direct target of miR-23a and its repression sensitizes CRC MSI cells to 5-FU [374].

Preliminary evidence was also obtained on the role of miR-221, miR-222, miR-let-7c, miR-638, miR-187 and miR-10b in CRC progression and metastasis formation. In particular, miR-221 and miR-222 regulate the activation of NF-κB and STAT3 in human CRC cell lines via *RelA* mRNA targeting; both factors are involved in the development and progression of CRC when constitutively activated [375].

The miRNA let-7c is downregulated in primary tumor tissues. Ectopic expression of let-7c in highly metastatic Lovo CRC cells significantly suppressed cell migration and invasion *in vitro* through the downregulation of *KRAS*, *MMP11* and *PBX3*. In contrast, the inhibition of let-7c in poorly metastatic HT29 cells increased cell motility and invasion through increased gene expression of its targets *KRAS*, *MMP11* and *PBX3* [376].

Many other miRNAs affect EMT by targeting EMT-associated genes, such as miR-638, which targets *SOX2* [377], miR-187 hinders SMAD-mediated EMT by directly suppressing the expression of *SOX4*, *NT5E* and *PTK6* [378], and miR-10b targets *HOXD10* [379]. The following table shows the main miRNAs involved in CRC progression, their respective target genes and altered molecular pathways (Table 3).

**Table 3:** miRNAs and their targets involved in CRC progression and metastasis.

Function	miRNA	Key Targets	Pathways Affected	Role in CRC	References
**EMT and Metastasis**	**miR-20a**	*SMAD4*	EMT	Promotes migration, invasion, and EMT; upregulated in CRC	[203,338]
	**miR-92a**	*PTEN*, *RECK*	EMT	Induces EMT and promotes CRC invasion, migration, and cell growth	[291,339]
	**miR-106a**	*TGFBR2*	EMT	Promotes migration and invasion of tumor cells; upregulated in metastatic CRC	[354,355]
	**miR-15a/16**	*Cyclin B1*, *AP4*	EMT	Suppresses EMT, metastasis, and CRC progression; downregulated in CRC	[128,360]
	**miR-10b**	*HOXD10*	EMT	Promotes EMT and invasion in CRC	[379]
	**let-7c**	*KRAS*, *MMP11*, *PBX3*	EMT	Suppresses CRC migration and invasion; downregulated in CRC	[376]
	**miR-638**	*SOX2*	EMT	Suppresses EMT in CRC	[377]
	**miR-187**	*SOX4*, *NT5E*, *PTK6*	EMT	Suppresses EMT in CRC	[378]
	**miR-133a**	*CXCR4*, *RhoA*	EMT, cytoskeletal reorganization	Suppresses tumor growth and metastasis; downregulated in CRC	[108,365]
	**miR-34**	*Notch1*, *Jagged1*, *ZNF281*, *SIRT1*	EMT, E2F signaling, IL-6R/STAT3	Suppresses migration, invasion, and EMT; inhibits metastasis and cell proliferation; downregulated in CRC; modulates p53 and inflammation	[[330], [331], [332], [333], [334]]
**EMT, Inflammation and Immune Regulation**	**miR-155**	*Claudin-1*, *SOCS1*, *ZBTB2*, *AXIN1*, *TCF4*	EMT, JAK2-STAT3/NF-κB, WNT/β-catenin	Promotes migration, invasion, metastasis, and inflammation; upregulated in CRC; involved in tumor progression and metastasis	[[325], [326], [327], [328], [329]]
	**miR-200 family**	*ZEB1*, *ZEB2*, *PRRX1*, *PD-L1*, *SIP1*	EMT, immune suppression	Suppresses EMT and metastasis; downregulated in CRC; promotes immunosuppression via PD-L1	[211,[347], [348], [349], [350]]
**Inflammation and Immune Regulation**	**miR-146a-5p**	*SOCS1*, *ZBTB2*	JAK2-STAT3/NF-κB	Promotes tumor progression and metastasis via inflammatory cytokines; involved in exosome trafficking from CAFs	[327]
	**miR-221/222**	*RelA*	NF-κB, STAT3	Promotes CRC development and progression; regulates inflammatory signaling	[375]
**Inflammation Immune Regulation, Apoptosis, Autophagy, Cell Proliferation and Tumor Growth**	**miR-124**	*DNMT3B*, *STAT3*, *PTB1*	Autophagy, apoptosis, inflammatory response	Suppresses CRC proliferation, migration, invasion, and STAT3 signaling; downregulated in CRC	[[340], [341], [342], [343], [344]]
**Apoptosis, Autophagy, Cell Proliferation and Tumor Growth**	**miR-206**	*FMNL2*, *c-MET*, *TM4SF1*, *NOTCH3*	Cell cycle, apoptosis	Suppresses CRC proliferation, invasion, and metastasis; downregulated in CRC	[113,362,363]
	**miR-21-5p**	*PDCD4*, *TET1*	Apoptosis, migration, metastasis	Enhances CRC migration, intravasation, and metastasis; upregulated in CRC	[368,369]
	**miR-31**	*Beclin-1*, *ATG*, *DRAM*, *LC3*	Autophagy	Promotes proliferation, invasion, and radiosensitivity in CRC; overexpressed in CAFs	[352]
	**miR-145-5p**	*CX43*, *RAS, E2F5*, *BAG4*, *FMNL2*	Gap junctions, RAS signaling	Inhibits angiogenesis, proliferation, and invasion; regulates extracellular matrix remodeling	[[335], [336], [337],^353^]
	**miR-195-5p**	*YAP*	Hippo-YAP	Represses cell growth, colony formation, and invasion; downregulates the Hippo-YAP pathway	[122]
**Cell Proliferation and Tumor Growth**	**miR-181a**	*SRCIN1*, *WIF-1*	SRC, VEGF, Wnt	Promotes angiogenesis, motility, and invasion; correlates with advanced stage and metastasis	[357,358]
	**miR-320a/c**	*β-catenin*	WNT/β-catenin	Inhibits CRC cell proliferation and migration; downregulated in CRC	[366,367]
	**miR-166-5p**	*ITM3E*	PI3K/Akt	Promotes cell proliferation; involved in G-MDSC-induced CRC progression	[370]
**Cell Proliferation, Tumor Growth, Drug Resistance, MSI, Prognostic Value**	**miR-203**	*-*	–	Associated with poor survival; upregulated in liver metastases	[371]
	**miR-23a**	*PDK4*, *ABCF1*	Microsatellite instability (MSI)	Promotes CRC cell proliferation; associated with drug resistance and MSI	[373,374]

### 3.3 microRNAs in colorectal cancer chemo- and radioresistance

Chemoresistance refers to the ability of tumor cells to withstand the effects of chemotherapy, leading to reduced treatment effectiveness, treatment failure, and, ultimately, the progression of the disease. Tumor cells develop chemoresistance through various mechanisms, including overexpression of ABC transporters and efflux of chemotherapeutic drugs, the overexpression of thymidylate synthase, the overexpression of anti-apoptotic proteins and resistance to apoptosis; these mechanisms enable tumor cells to resist apoptosis and survive treatment [380]. Such mechanisms are particularly active in CCSCs, which show strong resistance to chemotherapy and are the main cause of CRC recurrence [381].

Several molecular pathways are particularly associated with CRC chemoresistance due to their roles in cell survival, proliferation, and drug response. The multidrug resistance (MDR) pathway is currently responsible for the low effectiveness of chemotherapeutic agents. One of the key characteristics of CRC cells exhibiting MDR is the overexpression of the insulin-like growth factor type I receptor (*IGF-IR*). Suppressing *IGF-IR* leads to the inhibition of the PI3K/Akt signaling pathway, which in turn downregulates Nrf2-ARE-dependent transcriptional activity. This leads to a reduced activity of the multidrug resistance-associated protein-2 (*MRP-2*) promoter, limiting *MRP-2* expression and contributing to the reversal of chemoresistance [382]. Leptin is a pluripotent cytokine secreted by adipocytes and involved in the regulation of appetite and energy balance in the brain. Bartucci M. et al., found that obesity and increased leptin levels could counteract the cytotoxic effect of 5-FU promoting the growth and survival of CCSCs [383]. Apoptosis resistance is another strategy adopted by CRC cells for chemoresistance. For instance, the Human Ring-Finger homologous to Inhibitor of apoptosis protein type (*hRFI*) gene is involved in the inhibition of death receptor-mediated apoptosis in CRC cells. In a study by Konishi T et al., CRC cells were stably transfected with *hRFI*. The overexpression of *hRFI* resulted in cellular resistance to 5-FU through the inhibition of the mitochondrial apoptotic pathway, the upregulation of *BCL-2* and *BCL-X*, and the activation of *NF-kB* [384]. Moreover, elevated expression of thiamine synthase, *BCL-2*, *BCL-XL* and *Mcl-1* have been related to 5-FU resistance [385]. The tryptophan-aspartate repeat domain 43 (*WDR43*) is highly expressed in CRC tissues and its overexpression is associated with poor prognosis. WDR43 increases the ubiquitination of p53 by MDM2 through binding to RPL11. *WDR43* suppression significantly inhibits cell growth and enhances the effect of oxaliplatin chemotherapy both *in vitro* and *in vivo* [386]. The sex-determining region Y-box2 (*SOX2*), a master regulator of embryonic and induced pluripotent stem cells, sustains CSCs and plays an important role in tumor initiation and aggressiveness. A study showed that SOX2 promotes chemoresistance through the transcriptional activation of *ABCC2* expression. Specifically, SOX2 interacts with β-catenin and Beclin1 and increases their nuclear expression and transcriptional activity. Overexpression of β-catenin or Beclin1, in turn, promotes the expression of *ABCC2*, which, together with *Beclin1* and *SOX2*, influences chemoresistance, stemness and EMT in CRC [387].

As miRNAs regulate signaling pathways involved in chemoresistance, their altered expression may affect cellular sensitivity to chemotherapeutic agents. Indeed, numerous studies have shown that miRNAs contribute to drug resistance by modulating mechanisms and pathways associated with cell survival [388]. Slattery ML et al. performed an analysis of miRNAs and apoptosis-related genes on 217 CRC and normal tissues. Several miRNAs were identified as involved in the regulation of *BIRC5*, *CTSS* and *CSF2R*, all genes associated with apoptosis. Specifically, the authors demonstrated that *BIRC5* could be a potential target of miR-145-5p, miR-150-5p, miR-195-5p, and miR-650; *CSF2RB* a target of miR-92a-3p; *CTSS* a target of miR-20b-5p and miR-501-3p [389].

The miRNA-mediated dysregulation of genes involved in double-strand break (DSB) repair also contributes to the promotion of chemoresistance mechanisms [390]. For instance, a study investigating the co‐regulatory networks of tumor suppressor genes, oncogenes, and miRNAs occurring in CRC revealed that the overexpression of miR-17, miR-425 and miR-92 was significantly associated with up-regulation of BRCA1, counteracting the usually observed downregulation of genes involved in the mismatch repair pathway, including *MLH1*, *MSH2* and *MSH6* [391]. In line with these findings, other research groups have also investigated the role of antioxidant mechanisms in the occurrence of CRC chemoresistance. In this context, they demonstrated that the epigenetic regulation of glutathione (GSH) homeostasis is another mechanism that may induce the acquisition of drug resistance [392]. Specifically, miRNAs involved in the GSH homeostasis, such as miR-18a [287] or miR-214 [393] may influence the sensitivity of tumor cells to various therapeutic approaches.

miR-195-5p and miR-497-5p are downregulated in CRC tissues and have been widely studied in the context of drug resistance. HCT116 and RKO cells with MSI/P53 wild-type had increased sensitivity to oxaliplatin following transfection with miR-195-5p and miR-497-5p mimics [394]. Low miR-497 expression was strongly correlated with clinical stages and lymph node metastases. Furthermore, Ras suppressor kinase 1 (*KSR1*), a known oncogene overexpressed in human CRC samples, was identified as a direct target of miR-497. Overexpression of miR-497 in SW1116 CRC cells inhibited cell proliferation, migration and invasion and increased chemosensitivity to 5-FU, whereas forced expression of *KSR1* had the opposite effect [120]. In addition, miR-497 by targeting *IGF1-R* promotes inhibition of cell proliferation and invasion and promotes apoptosis induced by several stimuli, including the chemotherapeutic drugs cisplatin and 5-FU [117]. miR-497 was also found to be downregulated in the multidrug-resistant human gastric cancer cell line SGC7901/vincristine (VCR) and in the multidrug-resistant human lung cancer cell line A549/cisplatin (CDDP). In these models, the downregulation of miR-497 correlates with the upregulation of BCL2 protein, one of its direct targets. Thus, miR-497 could play a role in MDR through modulation of apoptosis by targeting *BCL2* [395]. Moreover, *BCL2* is a direct target of miR-195 and the overexpression of this miRNA in HT29 and LoVo cells promotes cell apoptosis and suppresses tumorigenicity [123]. In Dox-resistant CRC lines HT29/DOX and LOVO/DOX, miR-195 was significantly downregulated. Knockdown of miR-195 in HT29 and LOVO-sensitive cells inhibited Dox cytotoxicity, whereas overexpression of miR-195 sensitized Dox-resistant cells by targeting *BCL2L2* [124].

miR-125 is down-regulated in both colon cancer tissue and colon cancer cell lines demonstrating a tumor suppressor role; indeed, its overexpression inhibited cell proliferation and induced apoptosis in colon cancer cells. Overexpression of miR-125 leads to the repression of apoptosis, as the anti-apoptotic genes *BCL2, BCL2L12* and *Mcl-1* are direct targets of this miRNA [396]. Some studies have demonstrated the role of this miRNA in FOLFOX therapeutic efficacy. Notably, the FOLFOX regimen, consisting of the combination of 5-FU, leucovorin and oxaliplatin, is effective for the treatment of CRC [397]. However, the circRNA circ_0032833 was found significantly up-regulated in FOLFOX-resistant CRC and associated with drug resistance. Furthermore, circ_0032833 sequesters miR-125-5p, preventing its tumor-suppressing action. Among the targets of miR-125-5p, Musashi1 (*MSI1*) appears to be involved in 5-FU and oxaliplatin sensitization in FOLFOX-resistant CRC cells [398]. Another study demonstrated that the activation of the CXCL12/CXCR4 axis promotes EMT and the upregulation of miR-125b in CRC cells. Consequently, miR-125b promotes EMT, tumor invasion and *CXCR4* expression, thus generating a positive feedback that also involves the Wnt/β-catenin signaling since *APC* appears to be targeted by miR-125b. miR-125b also appears to confer resistance to 5-FU in CRC, probably through increased autophagy [399]. All these data suggest the dual role of miR-125, with some subtypes acting as tumor suppressor miRNAs and others as tumor-promoting ones [400].

The cluster miR-143/145 is often downregulated in CRC cells compared to normal colon epithelia. Restoration of miR-143 and miR-145 in CRC cells reduced proliferation, migration and chemoresistance [86]. miR-145 seems to sensitize LS174T cells to 5-FU by repression of Fli-1 [401]. CBR3-AS1 lncRNA is upregulated in CRC tissues and cell lines and correlates with poor prognosis and adverse clinicopathological features of CRC patients. Furthermore, it was observed that CBR3-AS1 promotes resistance to oxaliplatin in CRC cells by sponging and inhibiting miR-145 [402]. Stable expression of miR-143 decreases viability and increases cell death in CRC cells treated with 5-FU, probably through the modulation of pathways regulated by the extracellular protein kinase 5/NF-kB [100]. Hexokinase 2 (*HK II*) encodes for a limiting enzyme of glutamine metabolism and is responsible for the dysregulation of glycolysis in tumors. *HK II* is overexpressed in CRC and positively correlates with 5-FU resistance. miR-143, which is significantly downregulated in 5-FU-resistant CRC patients and colon cancer cells, targets *HK II*. The overexpression of miR-143 inhibits the rate of glycolysis by directly targeting *HK II*, leading to the resensitization of 5-FU-resistant colon cancer cells [403]. By analyzing miRNA expression in both 5FU-sensitive and 5FU-resistant DLD-1 cell lines, as well as in their corresponding extracellular microvesicles (MVs) before and after 5-FU treatment, it was found that miR-34a and miR-145 were actively secreted via MVs in both cell types. This suggests that these miRNAs may play a role in cellular communication and possibly in the development of chemoresistance [404].

Besides its mutual action with miR-145, miR-34a is down-regulated in 5-FU-resistant DLD-1 cells when compared with sensitive parental DLD-1 clones. *SIRT1*, a miR-34a target, is associated with drug resistance and is up-regulated in 5-FU-resistant cells. Ectopic expression of miR-34a in resistant cells attenuates 5-FU resistance through the down-regulation of *SIRT1* and *E2F3* [405]. In addition, mutations affecting p53 are important determinants of chemoresistance in CRC. Leucine-rich pentatricopeptide repeat-containing protein (*LRPPRC*) is a key downstream functional factor of p53 that can bind mRNA of ATP-binding cassette subfamily B member 1 mRNA 1 (*MDR1*), increasing its stability and protein expression. In normal cells, miR-34a represses *LRPPRC,* reducing *MDR1* expression. However, in p53 mutated cells, the accumulation of LRPPRC and MDR1 promotes drug resistance. To corroborate these findings, p53 mutated cells treated with gossypol-acetic acid (GAA), a specific inhibitor of LRPPRC, showed a reduced chemoresistance [406]. miR-34a was also found significantly downregulated in CRC clinical samples obtained from oxaliplatin-resistant patients and in multidrug-resistant CRC cells. Ectopic expression of miR-34a resensitized multidrug-resistant HCT-8/OR cells to oxaliplatin treatment, whereas miR-34a inhibition increased oxaliplatin resistance in chemoresistant HCT-8 cells. In these models, the mRNA of ornithine decarboxylase 2 (*OAZ2*) enzyme is targeted by miR-34a; therefore, the suppression of miR-34a/OAZ2 signal expression by chemotherapeutic agents increases the activation of MDR-associated ATP-binding cassette (ABC) transporters and anti-apoptosis pathways, thus leading to the development of MDR in CRC models [407].

As previously described, the miR-17/92 cluster is upregulated in CRC. miR-19b-3p expression was evaluated in 211 colon cancer patients, revealing its overexpression in patients with poor prognosis. Moreover, miR-19b-3p mediates resistance to oxaliplatin-based chemotherapy via *SMAD4* [408]. Exosomal miR-19b has been identified as a key contributor to oxaliplatin resistance in cancer cells. Inhibition of its secretion using GW4869, a pharmacological agent known to block exosome release, enhances the sensitivity of SW480 cells to oxaliplatin. This suggests that targeting exosomal pathways, specifically miR-19b, could be a promising strategy for overcoming chemoresistance and improving therapeutic efficacy in oxaliplatin-resistant cancers. By disrupting exosomal signaling, the potential for re-sensitizing resistant cancer cells to treatment becomes a viable approach for enhancing the effectiveness of chemotherapy [409]. By evaluating miRNA expression profiles in CRC patients, comparing a cohort of 295 chemosensitive and chemoresistant patients, miRNA-17-5p expression was found to be increased in the chemoresistant group. In addition, overexpression of miR-17-5p promoted the invasiveness and MDR of COLO205 via *PTEN* targeting [180]. Although miR-20b is generally up-regulated in CRC, a study reported its downregulation in 5-FU-resistant compared to 5-FU-sensitive tissues and cells. Restoration of miR-20b resensitizes 5-FU-resistant HCT116 by inducing apoptosis and repressing the expression of its targets *ADAM9* and *EGFR* [410].

The myo-miRNAs miR-206 and miR-133, often downregulated in CRC, also appear to play a role in chemoresistance. miR-206 was downregulated in 5-FU resistant CRC lines compared to their parental cell lines and this downregulation promotes drug resistance. The resistance conferred by the downregulation of miR-206 might depend on the increase of its target *Bcl-2* [411]. As regards miR-133b, this is a tumor suppressor miRNA in CRC. Indeed, a study demonstrated that miR-133b is downregulated in CRC spheroids, which are enriched in CSCs and show stem cell-like characteristics and high chemoresistance. Overexpression of miR-133b reduced CRC stemness and abrogated chemoresistance to 5-FU and oxaliplatin. These effects may depend on the role of miR-133b in regulating its direct target disruptor of telomeric silencing 1-like (*DOT1L*), an exclusive H3K79 methyltransferase important for stem cell gene modification [412].

In three oxaliplatin-resistant CRC lines, HT29, RKO, and HCT116, miR-203 was found to be up-regulated. The downregulation of miR-203 sensitized chemoresistant CRC cells to oxaliplatin. Moreover, ATM, a primary mediator of DNA damage response, is targeted by miR-203 and stable knockdown of *ATM* is associated with oxaliplatin resistance in chemosensitive CRC cells [413]. As mentioned in other sections, the lncRNA HOTAIR is upregulated in CRC tissues compared to adjacent control tissues and downregulates miR-203a-3p in CRC *in vitro* models. HOTAIR promotes the proliferation and drug resistance of CRC cells and the overexpression of miR-203a-3p in CRC cell lines inhibits cell proliferation and reduces chemoresistance [58].

miR-192/miR-215 expression levels were decreased in clinical colon cancer specimens compared with adjacent normal tissues of the same patients [414]. In CRC cells, miR-192 and miR-215 bind *TYMS*, one of the specific targets of fluoropyrimidine-based chemotherapies. Cell proliferation and S-phase cells are reduced by overexpression of miR-192/215. Consequently, the effects of S-phase-specific drugs are attenuated. These results suggest that mechanisms other than *TYMS* overexpression are essential for directing 5-FU resistance [415]. In patients treated with fluoropyrimidine-based chemotherapies, the miR-200 family seems to influence survival. For instance, high levels of miR-200a, miR-200c, miR-141, or miR-429 were correlated with longer overall and disease-free survival. In particular, high miR-429 levels result in the inhibition of CRC cell invasion after 5-FU treatment [416]. Table 4 lists the miRNAs related to chemoresistance acquisition identified so far (Table 4).

**Table 4:** miRNAs and their targets involved in CRC chemoresistance.

miRNA	Key Targets	Pathways Affected	Role in CRC	References
**miR-145-5p**	*BIRC5*, *Fli-1*	Apoptosis, Drug resistance	Sensitizes CRC cells to 5-FU by repression of Fli-1; potential target of BIRC5; downregulated in CRC and associated with oxaliplatin resistance	[389,401,402]
**miR-150-5p**	*BIRC5*	Apoptosis	Regulates apoptosis by targeting BIRC5; downregulation linked to CRC progression	[389]
**miR-195-5p**	*BIRC5*, *BCL2*, *BCL2L2*, *YAP*	Apoptosis, Drug resistance, Hippo-YAP	Downregulated in CRC; sensitizes cells to oxaliplatin and 5-FU by targeting BCL2 and BCL2L2; promotes apoptosis; overexpression inhibits tumorigenicity; associated with 5-FU resistance in HT29/DOX and LOVO/DOX cells	[123,124,389,394]
**miR-650**	*BIRC5*	Apoptosis	Potential regulator of apoptosis by targeting BIRC5	[389]
**miR-92a-3p**	*CSF2RB*	Apoptosis	Targets CSF2RB; involved in apoptosis regulation	[389]
**miR-20b-5p**	*CTSS*, *ADAM9*, *EGFR*	Apoptosis, EMT	Downregulated in 5-FU-resistant CRC cells; resensitizes CRC cells to 5-FU by inducing apoptosis and repressing ADAM9 and EGFR	[389,410]
**miR-501-3p**	*CTSS*	Apoptosis	Potential regulator of CTSS and apoptosis in CRC	[389]
**miR-497-5p**	*KSR1*, *BCL2*, *IGF1-R*	Apoptosis, Drug resistance, EMT	Downregulated in CRC; sensitizes cells to oxaliplatin and 5-FU; promotes apoptosis by targeting IGF1-R and BCL2; inhibits EMT and cell proliferation; enhances chemosensitivity	[117,120,394,395]
**miR-125-5p**	*MSI1*, *BCL2*, *Mcl-1*, *CXCR4*	Apoptosis, EMT, Autophagy	Tumor suppressor in CRC; downregulation leads to resistance to FOLFOX; targets BCL2, BCL2L12, and Mcl-1; modulates CXCR4 and APC signaling; also involved in drug resistance and autophagy	[396,[398], [399], [400]]
**miR-34a**	*SIRT1*, *E2F3*, *OAZ2*	Apoptosis, Drug resistance, EMT	Downregulated in 5-FU-resistant CRC cells; targets SIRT1, E2F3, and OAZ2; sensitizes multidrug-resistant CRC cells to 5-FU and oxaliplatin treatment	[[405], [406], [407]]
**miR-19b-3p**	*SMAD4*	Apoptosis, Drug resistance	Mediates oxaliplatin resistance by targeting SMAD4; overexpressed in patients with poor prognosis; inhibition of its exosomal secretion enhances oxaliplatin sensitivity	[408,409]
**miR-17-5p**	*PTEN*	Apoptosis, Drug resistance	Upregulated in chemoresistant CRC; promotes invasiveness and multidrug resistance (MDR) by targeting PTEN	[180]
**miR-143**	*HK II*	Glycolysis, Apoptosis, Drug resistance	Downregulated in CRC and 5-FU-resistant CRC cells; inhibits glycolysis by targeting HK II, resensitizes cells to 5-FU; inhibits proliferation and migration	[86,100,403]
**miR-206**	*Bcl-2*	Apoptosis, Drug resistance	Downregulated in CRC; promotes drug resistance by targeting Bcl-2	[411]
**miR-133b**	*DOT1L*	Stemness, Chemoresistance	Tumor suppressor miRNA; downregulated in CRC; reduces CRC stemness and abrogates chemoresistance to 5-FU and oxaliplatin by targeting DOT1L	[412]
**miR-203**	*ATM*, *HOTAIR*	DNA damage response, Drug resistance	Upregulated in oxaliplatin-resistant CRC lines; downregulation sensitizes cells to oxaliplatin by targeting ATM	[375,413]
**miR-192/215**	*TYMS*	S-phase, Drug resistance	Downregulated in CRC; modulates TYMS expression, which affects the efficacy of fluoropyrimidine-based chemotherapy	[414,415]
**miR-200 family**	*-*	EMT, Drug resistance	Associated with better prognosis in CRC patients treated with fluoropyrimidine-based chemotherapy; high levels correlate with longer survival	[416]

Besides chemotherapy, radiotherapy is another major treatment for unresectable or drug-resistant tumors, especially CRC. However, neoplastic cells can also acquire resistance to radiation exposure by developing a radioresistant phenotype through the modulation of various mechanisms, including autophagy, apoptosis, cell cycle control, ROS pathways, cancer stem cells (CSCs) and epithelial-mesenchymal transition (EMT) [417]. Similarly to what was described for the acquisition of resistance to chemotherapeutics, miRNAs may also act as modulators in cell signaling pathways that confer radioresistance [418].

For instance, it has been shown that miR-7-5p, which targets KLF4, is reduced in cancerous tissues of CRC patients radiotherapy resistant and that the miR-7-5p/KLF4 axis can induce radiosensitivity [419]. Sun T and colleagues suggested that miR-19b inhibition could enhance the efficacy of radiotherapy in CRC cells [194]. miR-195 can increase the radiosensitivity of CRC cells by targeting *CARM1* [420]. miR-185 can enhance radiosensitivity in CRC by targeting *IGF1R* and *IGF2* [421]. Circ-ACAP2 may promote CRC progression and radioresistance, in part by sponging miR-143-3p, which in turn modulates Wnt/β-catenin signaling [422]. miR-106b could induce cell radioresistance by directly targeting *PTEN* and *p21* [423]. Long noncoding RNA SP100-AS1 induces radioresistance in CRC by sponging of miR-622 which targets ATG3 and influences autophagy activity [424]. A study suggested that the circulating miRNAs miR-506-3p and miR-140-5p may have roles as biomarkers of radiosensitivity as they have higher expression levels in radiosensitive patients than in radioresistant patients [425]. The restoration of miR-1 promotes the expression of Bax and E-cadherin and decreases the expression of BCL2, MMP2 and MMP9, apparently impairing the invasion and migration of CRC cells in synergy with radiotherapy [426]. miR-222 and miR-155 could promote radioresistance in CRC by targeting *PTEN* and *FOXO3a*, respectively [427]. miR-29a may regulate the radiosensitivity of CRC cells by targeting *PTEN* [428]. miR-124 can radiosensitize CRC cells by targeting *PRRX*, an EMT inducer and regulator of stemness [429]. miR-378a-5p could resensitize CRC cells to radiotherapy by modulating the LRP8/β-catenin axis [430]. miR-1226-5p is involved in CRC radioresistance and through *IRF1* suppression activates M2 macrophages and induces TGF-β secretion [431]. *ATG12* and *LC3* are overexpressed in radioresistant CRC samples and miR-214 can promote radiosensitivity by inhibiting ATG12-mediated autophagy [393]. *ATG12* is also a target of miR-93, which in turn is sponged by the long non-coding RNA HOTAIR. Knockdown of HOTAIR increases radiosensitivity by modulating the miR-93/ATG12 axis [432]. Exosome-mediated transfer of miR-93-5p from CAFs to CRC cells can confer radioresistance through downregulation of FOXA1 and upregulation of TGFB3 [433]. Similarly, miR-590-3p transfer via CAFs-derived exosomes was found to enhance radioresistance in CRC through positive regulation of the PI3K/Akt signaling pathway [434]. Table 5 lists the miRNAs related to radioresistance acquisition identified so far (Table 5).

**Table 5:** miRNAs and their targets involved in CRC radioresistance.

miRNA	Key Targets	Pathways Affected	Role in CRC	References
**miR-7-5p**	*KLF4*	Stemness and radioresistance	Antitumor function in the regulation of CSC properties and radiosensitivity	[419]
**miR-19b**	*FBXW7*	Stemness and radioresistance	Modulation of the FBXW7/Wnt/β-catenin axis	[194]
**miR-195**	*CARM1*	Apoptosis and radioresistance	Downregulated in CRC, inhibits the expression of CARM1 which in turn regulates the expression of p53 and NF-κB involved in radiosensitivity	[420]
**miR-185**	*IGF1R* and *IGF2*	Apoptosis and radioresistance	Upregulation enhances radiosensitivity by targeting IGF1R and IGF2	[421]
**miR-143-3p**	*FZD4*	Progression and radioresistance	Modulation of the Wnt/β-catenin signaling by circ-ACAP2/miR-143-3p/FZD4 axis	[422]
**miR-106b**	*PTEN* and *p21*	Cell proliferation, tumour growth and radioresistance	Upregulation downregulates PTEN and p21 and subsequently enhances radioresistance.	[423]
**miR-622**	*ATG3*	Autophagy and radioresistance	Downregulated as sponged by SP100-AS1, affects autophagic activity by targeting ATG3 and contributes to radioresistance	[424]
**miR-506-3p and miR-140-5p**	–	–	Circulating biomarkers of radiosensitivity	[425]
**miR-1**	*BCL2*, *MMP2* and *MMP9*	Apoptosis and radioresistance	Downregulated in CRC, enhances radiosensitivity by inducing cell apoptosis	[426]
**miR-222**	*PTEN*	Cell proliferation, apoptosis inhibition, cell invasion and radioresistance	Upregulated, mediates radioresistance via PI3/Akt pathway	[427]
**miR-155**	*FOXO3a*	Cell proliferation, apoptosis inhibition, cell invasion and radioresistance	Upregulated, mediates radioresistance via PI3/Akt pathway	[427]
**miR-29a**	*PTEN*	Cell proliferation, tumour growth and radioresistance	Radiosensitivity regulation by targeting the PTEN gene	[428]
**miR-124**	*PRRX*	EMT, stemness regulation and radioresistance	Downregulated in CRC, enhances radiosensitivity by targeting PRRX	[429]
**miR-378a-5p**	*LRP8*	Cancer development and progression, radioresistance	Downregulated in CRC, regulates radioresistance via modulation of the Wnt/β-catenin pathway	[430]
**miR-1226-5p**	*IRF1*	EMT, migration, invasion, and tumor growth	In radioresistant CRC promoted EMT by targeting IRF1	[431]
**miR-214**	*ATG12*	Autophagy and radioresistance	Modulation of radioresistance by targeting ATG12	[393]
**miR-93**	*ATG12*	Apoptosis, autophagy and radiosensitivity	Modulation of radioresistance by targeting ATG12	[432]
**miR-93-5p**	*FOXA1*	Apoptosis, cell proliferation and radioresistance	Modulation of TGF-β signaling pathway and of radioresistance by targeting FOXA1.	[433]
**miR-590-3p**	*CLCA4*	Tumor growth	Enhances radioresistance through positive regulation of the CLCA4-dependent PI3K/Akt signaling pathway.	[434]

## 4 Role of circulating microRNAs in colorectal cancer

Notably, miRNAs are widely used as effective biomarkers for different diseases, including cancer. Several studies have demonstrated the diagnostic and prognostic value of differentially expressed miRNAs detected in both tissue and liquid biopsy samples obtained from CRC patients and healthy controls [435,436]. More recently, circulating miRNAs were proposed as non-invasive and reliable biomarkers for tumor diagnosis and patients’ follow-up due to their stability, the low costs of the analysis and the possibility of repeat sampling multiple times during the treatments [437].

Several studies have investigated the diagnostic accuracy of miRNAs and circulating miRNAs in CRC in terms of sensitivity, specificity, odds ratio (OR) and area under the ROC curve (AUC). Examples are miR-21-5p [[438], [439], [440], [441], [442]], miR-1290 [443], miR-210 [438,441], miR-378e [444], miR-1246 [445], miR-92a-1 [446], miR-320d [443], miR-15b [442] or miR-150-5p [447] which showed AUC values ranging from 0.7 to > 0.95 thus demonstrating significant potential as diagnostic biomarkers for the detection of CRC. Moreover, the comparison between protein biomarkers and the circulating levels of miR-133a, miR-574-3p and miR-27a has demonstrated a better sensitivity of these latter biomarkers (AUC = 0.736 (0.639–0.834) for CA19.9 and 0.88 (0.814–0.946 for CEA), both when analyzed alone or in combination (AUC = 0.974 (0.948–1.000) for miR-133a, 0.975 (0.948–1.000) for miR-574-3p and 0.904 (0.849–0.958) for miR-27a) [448]. Other studies have also investigated the potential prognostic role of circulating miRNAs. In particular, the serum levels of miR-93-5p could play a prognostic role for early disease recurrence (p = 0.035) in CRC patients with liver metastases who showed higher levels in metastatic vs non-metastatic tumors (p < 0.001) [449]. Also circulating miR-618 has been suggested as a possible prognostic biomarker in metastatic colon cancer since its up-regulation is associated with a better prognosis (overall survival (OS) of 21 months) compared to patients with low miR-618 expression (OS of 16 months; HR = 0.51, 95 % CI: 0.30–0.86, *p* = 0.012) [450]. The overexpression of miR-326, miR-27b and miR-148a was associated with low PFS, while miR-326 was associated with low OS [451]. Circulating miRNAs may also serve as predictive biomarkers for treatment response, offering a non-invasive tool to anticipate the efficacy of specific therapeutic strategies. For instance, Zhang J and colleagues proposed a profile of five serum miRNAs (miR-20a, miR-130, miR-145, miR-216 and miR-372) as a biomarker to predict CRC chemosensitivity [452]. High expression of miR-345 was associated with a non-response to treatment with irinotecan and cetuximab [453]. It has been observed that an increased serum level of miR-155 after surgery and chemotherapy is a sign of chemoresistance in CRC, and elevated levels of miR-155, miR-200c and miR-210 imply local recurrence and distant metastases as well as a poor prognosis [454]. Circulating miR-20b-5p, miR-29b-3p and miR-155-5p were significantly associated with PFS and OS as well as with response to bevacizumab in patients with metastatic CRC [455].

Moreover, circulating miRNAs may play a significant role in the development of CRC chemoresistance by modulating gene expression and influencing various cellular processes related to drug response. A study investigated circulating miRNAs as biomarkers of chemoresistance for oxaliplatin therapy in CRC patients. In particular, six miRNAs, miR-100, miR-92a, miR-16, miR-30e, miR-144-5p and let-7i, were verified as significantly and consistently downregulated (>1.5-fold, P < 0.05) in oxaliplatin-resistant patients. GO and KEGG pathway analysis showed that these miRNAs were able to modulate the RNA polymerase II transcription and the PI3K-AKT signaling pathway, AMPK signaling pathway and FoxO signaling pathway [456]. Jin G and colleagues selected 30 miRNAs that are aberrantly expressed during CRC progression based on previous microarray analyses. Subsequently, the expression levels of these miRNAs were assessed in oxaliplatin/5-FU-resistant CRC cell lines and in the corresponding secreted exosomes. Notably, miR-21-5p, miR-1246, miR-1229-5p, miR-135b, miR-425 and miR-96-5p were found up-regulated in exosomes obtained from the supernatant of resistant cells. Through GO and pathway prediction analysis, it was found that these miRNAs are involved in the PI3K-Akt, FoxO and autophagy signaling pathways. Therefore, targeting these miRNAs could promote chemosensitivity to oxaliplatin and 5-FU, representing a promising strategy for the treatment of resistant CRC [457]. Another study assessed the modulation of circulating miRNA levels in peripheral blood samples obtained from 77 5-FU-treated CRC patients. Differential expression of circulating miRNA levels was evaluated at three different time points: baseline, after 3 and after 6 months of treatment. Specifically, the expression levels of five miRNAs, miR-223-3p, miR-20a-5p, miR-17-5p, miR-19a-3p and miR-7-5p, and the expression of three proteins, PTEN, ERK and EGFR, were assessed. At baseline, CRC patients had significantly higher levels of circulating miRNAs than healthy controls. These levels decreased during 5-FU therapy and then increased significantly only in responder patients after 6 months. In particular, miR-19a-3p demonstrated a marked change in patients with elevated ERK, EGFR, and PTEN protein levels, showing a significant correlation with increased risk of disease recurrence and progression at the 6-month evaluation. This pattern suggests that miR-19a-3p could serve as a potential biomarker for early detection of aggressive disease behavior, particularly in patients with these specific molecular profiles [458]. In a study by Chen Q et al., the differential expression of circulating miRNAs from the serum of drug-responsive and drug-resistant patients was analyzed by microarray. Among the most significantly differentially expressed miRNAs between responders and non-responders, miR-221, miR-222, miR-122, miR-19a and miR-144 were selected for further validations in an independent cohort (N = 72). Notably, serum miR-19a levels were found to predict both intrinsic and acquired drug resistance [459]. miR-21-5p was frequently up-regulated in solid tumors, including CRC. The expression of miR-21-5p was found to be significantly up-regulated in the exosomes of CRC cells compared to normal human colon epithelial cells. Treatment of CRC cells with isolated exosomes or miR-21-5p mimic resulted in increased expression of genes involved in cell proliferation, invasion, and extracellular matrix degradation. These effects depended on miR-21-5p-mediated downregulation of its targets *PDCD4*, *TPM1* and *PTEN*. In particular, miR-21-mediated *PDCD4* silencing increases CRC resistance to 5-FU [311].

## 5 Conclusions

CRC is the fourth-deadliest cancer in the world and its incidence is constantly increasing worldwide. As highlighted in this review, miRNA expression profiles differ between normal mucosa and CRC tissue. The data here reported strongly support the role of miRNAs in CRC development and progression since miRNAs regulate cancer cell proliferation, migration, and invasion by modulating several molecular pathways, including Wnt/β-catenin, PI3K-AKT, RAS, MAPK, TGF-β and p53 signaling.

Several studies have also proposed miRNAs both as markers and therapeutic targets or for the development of novel RNA-based antitumor treatments. In this context, a better understanding of the role of miRNAs in CRC tumorigenesis and progression may provide new insights for non-invasive diagnostic tools for CRC screening and personalized therapy [460]. The present comprehensive review also highlights the role of miRNAs in mediating CRC drug resistance. These findings suggest the need for innovative *in vitro* and *in vivo* studies aimed at investigating the potential therapeutic application of miRNAs.

Despite these promising updates on miRNA research, several critical limitations remain in our understanding of the miRNA–CRC axis, which must be addressed to translate current knowledge into clinical impact. First, the context-dependent nature of miRNA activity remains a major challenge. A single miRNA can bind different targets and may act as a tumor suppressor in one setting and as an oncogene in another, depending on the cellular environment, the presence of specific cofactors, or even the cancer stage. This plasticity complicates therapeutic targeting and calls for more refined models that can account for tumor heterogeneity and dynamic miRNA–target interactions [461].

Moreover, as widely discussed in this review article, while many studies have identified dysregulated miRNAs in CRC through high-throughput profiling, relatively few have functionally validated these findings in relevant *in vivo* models. There is a significant gap between correlative studies and mechanistic investigations that clarify the downstream pathways affected by miRNAs, their upstream regulators, and the crosstalk with other molecular networks such as epigenetic modifications, immune response, and microbiota-host interactions.

Additionally, the therapeutic potential of miRNA-based interventions, though promising, is still constrained by delivery challenges, off-target effects, and the lack of tumor-specific targeting strategies. Most delivery systems used in preclinical models are not yet clinically feasible, and systemic administration of miRNA mimics or inhibitors may lead to unintended modulation of non-target tissues.

In the context of circulating miRNAs as non-invasive biomarkers, several limitations in the standardization of sample processing, the definition of normalization strategies, and the use of analytical platforms still exist. Specifically, circulating miRNA profiles are usually analyzed using RNA sequencing and microarray platforms and then validated through reverse transcription quantitative polymerase chain reaction (RT-PCR) or digital droplet PCR (ddPCR), with profound differences among these techniques. As regards miRNA profiling, microarray technology guarantees high throughput and multiplexing. However, conventional microarray technologies have a limited dynamic range and sensitivity [462]. SmallRNA sequencing by NGS is the most adopted method as it requires less starting material, allows the identification of miRNA isoforms and has the highest throughput [463]. Among the validation methods, despite RT-qPCR having low throughput compared to other techniques, it has advantages in terms of cost-effectiveness and speed [464]. When using RT-qPCR, it is important to normalize miRNA expression in order to reproduce data between studies, however, no stable endogenous controls have been identified yet. In contrast, ddPCR exhibits a higher tolerance to inhibitors than conventional RT-qPCR and allows an absolute quantification of miRNA expression [465,466]. Other technical issues related to miRNA quantification are due to pre-analytical factors, including appropriate sample volumes, sample handling, RNA extraction methods, quantification and normalization methods. The conflicting data on the expression levels of miRNAs in the different studies are partly due to the differences in these variables.

Finally, miRNAs involved in CRC chemo- and radioresistance are often studied in isolation, ignoring the complex interplay within the tumor microenvironment and the compensatory pathways that may undermine therapeutic efficacy. The dynamic response of miRNA expression to treatment further complicates their use as predictive markers, underscoring the need for longitudinal and integrative studies that combine transcriptomic, proteomic, and functional data.

Overall, all the findings here discussed highlight critical gaps in the current knowledge of miRNA-CRC axis; therefore, future research should prioritize the functional validation of miRNA–target interactions in clinically relevant models, the development of robust, specific delivery platforms for therapeutic use, the multi-omics integration to map miRNA-mediated networks and the development of consensus protocols for the clinical evaluation of circulating miRNAs as biomarkers.

## CRediT authorship contribution statement

**Federica Longo:** Writing – review & editing, Writing – original draft, Data curation, Conceptualization. **Giuseppe Gattuso:** Writing – original draft, Investigation, Formal analysis. **Graziana Spoto:** Writing – original draft, Investigation, Formal analysis. **Daria Ricci:** Writing – original draft, Investigation, Formal analysis. **Anastasia Cristina Venera Vitale:** Writing – original draft, Investigation, Formal analysis. **Alessandro Lavoro:** Investigation, Formal analysis. **Saverio Candido:** Investigation, Formal analysis. **Massimo Libra:** Writing – review & editing, Visualization, Supervision, Funding acquisition. **Luca Falzone:** Writing – review & editing, Writing – original draft, Supervision, Funding acquisition, Data curation, Conceptualization.

## Availability of data and materials

Not applicable.

## Ethics approval and consent to participate

Not applicable.

## Consent for publication

Not applicable.

## Funding

This work was supported in part by: 10.13039/501100000780European Union - NextGenerationEU through the Italian Ministry of University and Research under PNRR M4C2—Action 1.4—Call “Potenziamento strutture di ricerca e creazione di “campioni nazionali di R&S”—Project “National Center for Gene Therapy and Drugs based on RNA Technology” (CN00000041) to Professor Massimo Libra (CUP: E63C22000950006). The views and opinions expressed are those of the authors only and do not necessarily reflect those of the European Union or the European Commission. Neither the European Union nor the European Commission can be held responsible for them.

Prof. Luca Falzone was supported by the PIAno di inCEntivi per la RIcerca di Ateneo 2024/2026 - Linea di Intervento 1 “Progetti di ricerca collaborativa" (Project Code: BioEpiRes), and the Piano di incentivi per la ricerca di Ateneo 2024/2026 (Pia.ce.ri.), Linea di intervento 3 - Starting Grant (Project Code: ResCOr), 10.13039/501100004505University of Catania (Catania, Italy).

## Declaration of competing interest

The authors declare that they have no known competing financial interests or personal relationships that could have appeared to influence the work reported in this paper.
